# Targeting SKAP2 restores sperm motility and morphology through modulating mitochondrial organization and cytoskeletal remodeling

**DOI:** 10.1038/s41392-025-02513-3

**Published:** 2025-12-24

**Authors:** Shiming Gan, Lin Yin, Jiaming Zhou, Sisi Li, Shumin Zhou, Xiaotong Yang, Rui Liu, Xu Fan, Yangyang Li, Zhendong Yao, Jingshou Chen, Peiran Hu, Wenjing Xiong, Yuan Yuan, Yujiao Wen, Youjiang Li, Ge Jin, Jianzhong Sheng, Yuzhen Gao, Hefeng Huang, Chen Zhang

**Affiliations:** 1 Department of Reproductive Medicine, Center for Reproductive Medicine, the Fourth Affiliated Hospital of School of Medicine, and International School of Medicine, International Institutes of Medicine, Zhejiang University, Yiwu, China; 2 Institute of Reproduction and Development, Shanghai Key Laboratory of Reproduction and Development, Obstetrics and Gynecology Hospital, Fudan University, Shanghai, China; 3 Department of Urology & Andrology, Sir Run Run Shaw Hospital, Zhejiang University School of Medicine, Hangzhou, China; 4 Shandong Provincial Key Laboratory of Animal Cell and Developmental Biology, School of Life Sciences, Shandong University, Qingdao, China; 5 Institute of Reproductive Health, Tongji Medical College, Huazhong University of Science and Technology, Wuhan, China; 6 Department of Obstetrics and Gynecology, Union Hospital, Tongji Medical College, Huazhong University of Science and Technology, Wuhan, China; 7 School of Traditional Chinese Medicine, Shenyang Medical College, Shenyang, China; 8 Department of Clinical Laboratory, Sir Run Run Shaw Hospital, Zhejiang University School of Medicine, Hangzhou, Zhejiang China; 9 Institute of Medical Genetics and Development, Key Laboratory of Reproductive Genetics (Ministry of Education) and Women’s Hospital, Zhejiang University School of Medicine, Zhejiang, China; 10 Shanghai Key Laboratory of Female Reproductive Endocrine Related Diseases, Shanghai, China; 11 Asian Academy of Anti-aging Research and Translational Medicine (Shenzhen), Shenzhen, China

**Keywords:** Reproductive disorders, Developmental biology

## Abstract

Sperm motility and morphology are indispensable for sperm-egg interaction and successful fertilization. However, the RNA splicing mechanisms in an m6A-dependent manner regulating spermiogenesis-related genes remain poorly defined, and targeted therapy strategies to restore impaired sperm motility and morphology are lacking. In this study, we identify heterogeneous nuclear ribonucleoprotein R (hnRNPR) as a critical m6A-dependent splicing mediator. Pathogenic mutations in *HNRNPR* cause sperm motility decline, morphological abnormality, and male infertility in both humans and mice. Mechanistically, *Hnrnpr* mutation disrupts m6A-dependent splicing of *Skap2* pre-mRNA, thus impairing cytoskeletal structure and mitochondrial organization in sperm. Consistently, specific knockout of *Skap2* in male germ cells displays sperm abnormalities, which phenocopy those observed in humans and mice with *Hnrnpr* mutants, unveiling a functional hnRNPR-SKAP2 axis. Leveraging these insights, we developed a therapeutic strategy to restore sperm motility and morphology, relying on extracellular vesicle-mediated SKAP2 delivery to enter the efferent ductules of the testicles, which could promote sperm cytoskeletal remodeling and mitochondrial organization. Notably, the co-culture of extracellular vesicle SKAP2 with human and mouse sperms also significantly enhanced the sperm motility. Altogether, these findings identify hnRNPR as a pivotal regulator of m6A-mediated *Skap2* splicing during spermiogenesis and highlight extracellular vesicle SKAP2 as a promising therapeutic target for poor sperm quality and male infertility.

## Introduction

Reproductive capacity is essential for species continuity, yet infertility is rising worldwide and has become a critical public health issue with profound social and psychological impacts.^1^ Among its diverse causes, genetic variation and defective spermiogenesis represent major contributors to asthenoteratozoospermia,^2^ which is a complicated condition with severely reduced progressive sperm motility (<32%) and high frequency of morphologically abnormal spermatozoa (<4% normal forms).^3^ These combined functional and structural defects compromise the sperm’s ability to reach and fertilize the egg,^4^ therefore leading to conception failure even with assisted reproductive technologies. Despite its clinical significance, the genetic etiology of asthenoteratozoospermia remains incompletely understood. So far, only a small number of causative mutations have been reported, and the underlying molecular mechanisms by which these variants disrupt sperm motility and morphology are still unclear.

Male germ cell development is a highly orchestrated process in which RNA-binding proteins (RBPs) play pivotal roles, involving in RNA splicing, cell fate determination, and maintenance of cellular homeostasis.^5^ Alternative splicing, as a key post-transcriptional regulation mechanism, is essential for cell differentiation and development during spermatogenesis.^6^ This process is further fine-tuned by RNA modifications, among which N6-methyladenosine (m6A) is the most abundant internal mRNA modification in mammals.^7^ m6A has garnered considerable attention for its effect on RNA metabolism, including splicing, stability, and translation.^8,9^ Its dynamic regulation is mediated by “writers” (e.g., METTL3, METTL14, METTL16, WTAP), “erasers” (e.g., FTO, ALKBH5), and “readers” (e.g., YTH domain proteins, IGF2BPs, PRRC2A, and various heterogeneous nuclear ribonucleoproteins, hnRNPs).^10^ Compelling evidence underscores the indispensable role of m6A in male germ cell development. Conditional knockout of Mettl3 or Mettl14 depletes spermatogonial stem cells (SSCs),^11^ while Alkbh5-deficient mice exhibit pachytene arrest and impaired spermiogenesis.^12^ Meanwhile, m6A readers such as PRRC2A,^13^ YTHDC2,^14^ and YTHDF2^15^ are essential for meiotic process and spermatid maturation. Among the hnRNP family, loss of hnRNPC, which is a potential m6A reader, leads to azoospermia,^16^ implying the critical role of m6A-binding proteins in male fertility performance. Recently, hnRNPR was reported to stabilize ASCL1 transcripts in an m6A-dependent manner,^17^ however, its role in spermatogenesis and associated mechanism in m6A-modulated RNA splicing remain unknown.

Human sperm is unique in being the only cell type capable of functioning outside the organism, and its motility is essential for its fertilizing potential.^18^ Yet, restoring sperm motility remains a major challenge, especially when defects arise from genetic defects. Extracellular vesicle levels are markedly decreased in oligoasthenozoospermia, which is characterized by reduced sperm count and motility.^19^ Consistently, mouse models lacking vesicle-associated proteins also exhibit impaired male fertility.^20^ Intriguingly, Murdica et al. reported that extracellular vesicles from normozoospermic men could enhance sperm motility and promote subsequent activation and capacitation, whereas vesicles derived from men with asthenozoospermia could trigger impaired sperm quality.^21,22^ These findings suggest that proper supplementation with extracellular vesicles may counteract detrimental effects on sperm development. Recently, milk-derived extracellular vesicles (mEVs) have emerged as promising biomedical tools, serving both as disease biomarkers and therapeutic agents.^23^ These vesicles could transport proteins, lipids, metabolites, and nucleic acids into specific target cells.^24^ Building on this concept, we develop a novel therapeutic strategy using extracellular vesicles-SKAP2 (Src kinase-associated phosphoprotein 2) to ameliorate sperm motility. One well-characterized role of SKAP2 is being a cytoskeletal regulatory protein that plays a pivotal role in cell motility and signal transduction.^25^ Recent studies showed that SKAP2 also facilitates F-ACTIN assembly and asymmetric cytokinesis by interacting with nucleation-promoting factors such as WAVE2.^26,27^ Since F-ACTIN assembly is indispensable for proper sperm morphology and motility,^28–30^ SKAP2-enriched vesicles may represent a promising targeted approach to correct spermiogenesis defects and improve male fertility.

In this study, we reveal a previously unrecognized role of hnRNPR in male germ cell development and infertility. We identify pathogenic *HNRNPR* mutations linked to asthenoteratozoospermia and demonstrate that hnRNPR is indispensable for spermiogenesis. Mechanistically, hnRNPR regulates m6A-dependent alternative splicing in round spermatids. Along its downstream targets, we identify *Skap2*, a potential contributor for F-ACTIN assembly. Furthermore, we show that extracellular vesicles equipped with SKAP2 could rescue sperm motility decline and morphology defects by modulating sperm cytoskeletal remodeling and mitochondrial organization. Collectively, these findings not only uncover a novel mechanism whereby m6A-mediated splicing coordinates sperm differentiation, but also develop extracellular vesicles SKAP2-based intervention as a potential therapeutic strategy for male infertility.

## Results

### Mutations in the *HNRNPR* gene cause asthenoteratozoospermia and its association with male infertility

**Fig. 1:**
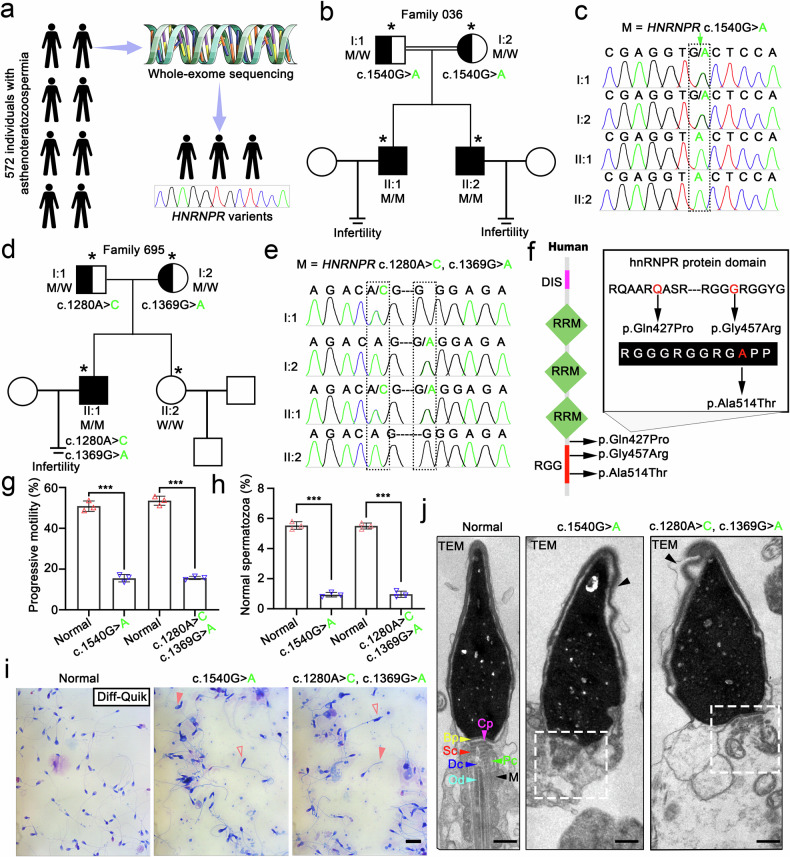
*HNRNPR* mutations cause asthenoteratozoospermia and male infertility. **a** Flowchart depicting the identification of human *HNRNPR* variants associated with asthenoteratozoospermia. **b**, **d** Pedigree analyses of two families harboring inherited *HNRNPR* mutations, identified by whole-exome sequencing (WES). Open squares and circles represent unaffected males and females, respectively, while filled black squares denote affected males carrying *HNRNPR* variants (NM_001102398.3). Sanger sequencing results are shown adjacent to each pedigree. Heterozygous variants in the parents confirm the nucleotide changes. Double lines indicate consanguineous marriages, and asterisks mark individuals who underwent WES. M: mutation; W: wild type. **c**, **e** Sanger sequencing confirming homozygous and compound heterozygous *HNRNPR* mutations in affected individuals. Three patients (II:1, II:2 in family 036; II:1 in family 695) inherited the variants from heterozygous parents (I:1 and I:2). Mutation sites are highlighted with dotted rectangles. **f** Amino acid and protein domain map showing the mutation locations within (p.Gly457Arg, p.Ala514Thr) or adjacent to (p.Gln427Pro) the RGG domain. Altered residues are marked in red within aligned sequences. **g**, **h** CASA of progressive motility (**g**) and the proportion of morphologically normal sperm (**h**) in healthy controls and patients. Data are shown as mean ± SD; statistical significance was assessed by two-sided Student’s *t*-test. *n* = 3 per group. **i** Representative Diff-Quik-stained human epididymal sperm smears from healthy individuals and patients. Sperm heads appear dark blue, acrosomes lavender, and midpieces/tails pale red. Hollow pink arrowheads indicate abnormally shaped sperm; solid pink arrowhead indicates a curved flagellum. Scale bar = 10 μm. **j** Transmission electron microscopy (TEM) images of sperm head ultrastructure. Black arrowheads indicate degenerating acrosomes, and white dotted lines outline structural defects in the sperm connecting piece. Bp basal plate, Cp capitulum, Sc segmented column, Pc proximal centriole, Dc distal centriole, M mitochondrion, Od outer dense fibers. Scale bar = 1 μm

From 2020 to 2025, we recruited a cohort of 572 males with asthenoteratozoospermia to identify novel pathogenic genes (Fig. 1a). Using a robust bioinformatic pipeline, we prioritized gene categories and variant types most likely involved in infertility. Within this cohort, we focused on three infertile males from one consanguineous and one non-consanguineous family. Clinical trio whole-exome sequencing (WES) combined with homozygosity mapping in the index family enabled the detection of loss-of-function (LoF) and deleterious nonsynonymous (DNS) variants. This analysis revealed a homozygous missense variant (c.1540 G > A) in *HNRNPR* in two brothers from the consanguineous family, and compound heterozygous missense variants (c.1280 A > C, c.1369 G > A) in the proband from the non-consanguineous family (Fig. 1b–e and Supplementary Table 1). Targeted Sanger sequencing validated these WES findings and demonstrated variant co-segregation within both families: the probands carried the respective *HNRNPR* variants, while both sets of parents were heterozygous carriers. In the second family, the proband’s healthy younger sister did not harbor either familial *HNRNPR* variant (Fig. 1c, e).

To further investigate the role of RNA-binding protein (RBP) variants in male infertility, we performed a comprehensive analysis of the underlying genetic landscape and identified 11 variants exclusively present in affected individuals, suggesting that RBP mutations deserve more attention in male infertility (Supplementary Fig. 1a). In the three patients harboring *HNRNPR* mutations, amino acid and domain mapping revealed that all variants were located within or adjacent to the RGG domain (Fig. 1f). Functional assessment using computer-assisted sperm analysis (CASA) showed that, although sperm concentration remained within the normal range, both progressive motility and the proportion of morphologically normal sperm were markedly reduced, consistent with a clinical diagnosis of asthenoteratozoospermia (Fig. 1g, h and Supplementary Table 2). Diff-Quik staining confirmed widespread morphological abnormalities, and transmission electron microscopy (TEM) revealed acrosome shedding and disorganized neck structures in spermatozoa carrying *HNRNPR* mutations (Fig. 1i, j). Collectively, these findings provide strong evidence that *HNRNPR* mutations could give rise to asthenoteratozoospermia.

### *Hnrnpr* mutations lead to asthenoteratozoospermia and male sterility in mice

Comparative amino acid sequence and phylogenetic analyses demonstrated that hnRNPR is highly conserved among vertebrates, with 99.06% sequence identity between human and mouse proteins (Supplementary Fig. 1b, c), suggesting that insights from the mouse model are highly relevant to human physiology. Thus, to investigate the role of hnRNPR in the male reproductive system and determine the specific stage of spermatogenesis at which it functions, we first examined its spatiotemporal expression across mouse organs and different developmental stages. Notably, *Hnrnpr* mRNA was abundantly expressed in the testis (Supplementary Fig. 1d). To further characterize its expression pattern during spermatogenesis, we reanalyzed published single-cell RNA-seq datasets. We found that the expression level of *Hnrnpr* was low in spermatogonia but reached peak in early round spermatids stage (Supplementary Fig. 1e). Consistently, immunofluorescence (IF) analysis of adult mouse testes showed that hnRNPR is predominantly presented in early round spermatids (Supplementary Fig. 1f, g).

**Fig. 2:**
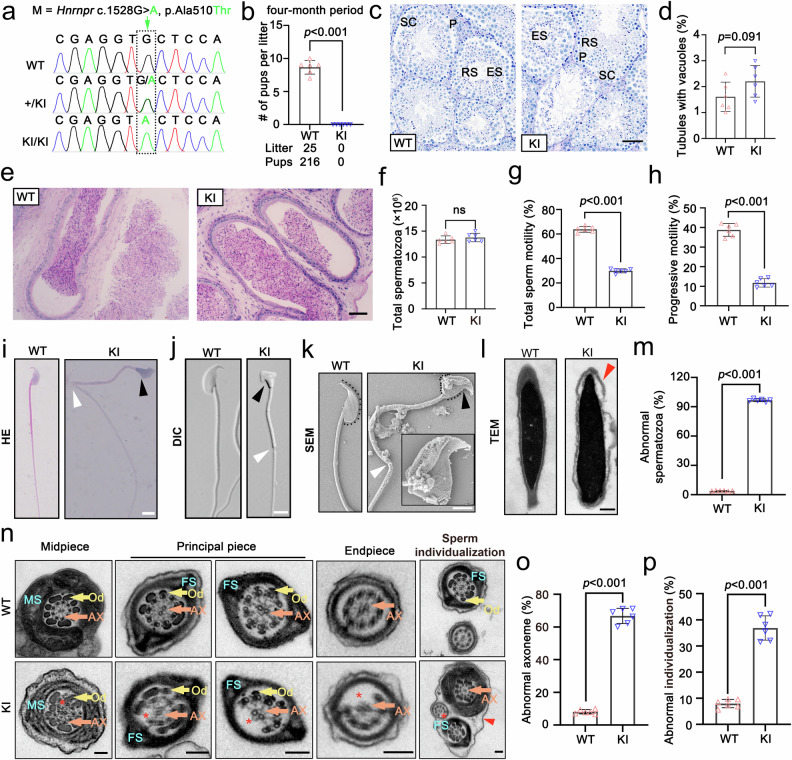
*Hnrnpr* mutations cause asthenoteratozoospermia in mice. **a** Sanger sequencing-based genotyping of WT and KI mice. **b** Fertility assessment over four months of mating between WT and KI mice. Data are mean ± SD; statistical significance was determined using a two-sided Student’s *t*-test. *n* = 6 per group. **c** Periodic acid-Schiff (PAS) staining showing normal seminiferous tubule morphology in both WT and KI testes. Scale bars: 50 μm. **d** Histogram of the proportion of abnormal seminiferous tubules in WT vs. KI mice. Data are mean ± SD; *P*-values from a two-sided Student’s *t*-test. *n* = 6 per group. **e** Histology of the epididymal cauda in WT and KI mice. Scale bars: 50 μm. **f** Sperm counts in WT and KI mice. Data are mean ± SD; no significant difference (ns) by two-sided Student’s *t*-test. *n* = 6 per group. **g**, **h** Quantitative analysis of total sperm motility (**g**) and progressive motility (**h**). Data are mean ± SD; *P*-values from a two-sided Student’s *t*-test. *n* = 6 per group. **i**–**k** Representative images of sperm from WT and KI mice, including H&E staining (HE), differential interference contrast (DIC), and scanning electron microscopy (SEM). Black arrowheads indicate abnormal sperm heads; white arrowheads indicate bent flagella. Scale bars: 5 μm. **l** Transmission electron microscopy (TEM) of sperm heads showing abnormal acrosomes (red arrowheads). Scale bars: 1 μm. **m** Histogram quantifying morphologically abnormal sperm (300 sperm per mouse). Data are mean ± SD; statistical significance by two-sided Student’s *t*-test. *n* = 6 per group. **n** TEM of sperm flagella (midpiece, principal piece, endpiece). Components: AX, axoneme; FS, fibrous sheath; Od, outer dense fiber; MS, mitochondrial sheath. Red asterisks indicate missing axonemal microtubule doublets; red arrowheads indicate defective sperm individualization (multiple sperm enclosed in one membrane). Scale bars: 200 nm. **o** Quantification of abnormal axoneme structures in midpiece, principal piece, and endpiece (100 segments per mouse). Data are mean ± SD; compared by two-sided Student’s *t*-test. *n* = 6 per group. **p** Quantification of defective sperm individualization (100 sperm per mouse). Data are mean ± SD; statistical significance assessed by two-sided Student’s *t*-test. *n* = 6 per group

To further elucidate the pathogenic mechanism through which *HNRNPR* mutations cause asthenoteratozoospermia, we generated a knock-in (KI) mouse model (Fig. 2a). Compared to wild-type (WT) mice, KI mice were identical in body size or testis-to-body weight ratio (Supplementary Fig. 2a–d). However, fertility testing revealed complete failure of offspring birth in *Hnrnpr*-mutant male mice (Fig. 2b and Supplementary Table 3). PAS staining showed no differences in the number of empty seminiferous tubules or total sperm counts between KI and WT group (Fig. 2c–f). However, CASA analysis revealed a remarkably reduced ability of both total and progressive sperm motility in KI mice, relative to control (Fig. 2g, h). Detailed morphological assessment using quantitative morphology software demonstrated that approximately 96.35% of sperm from *Hnrnpr* KI mice exhibited abnormal head and flagellum structures, compared with littermate controls (Fig. 2i–m). Transmission electron microscopy further revealed ultrastructural defects in sperm flagella, including absence of the canonical ‘9 + 2’ microtubule arrangement and multiple flagella enclosed within a single shared cell membrane (Fig. 2n–p). Collectively, these findings indicate that *Hnrnpr* KI mice reflected the key features of asthenoteratozoospermia observed in patients carrying *HNRNPR* mutations.

To further explore the specific role of hnRNPR in male germ cell development, we generated germ cell-specific hnRNPR conditional knockout mice (DDX4-Cre; *Hnrnpr*^flox/Del^, herein designated DDX4-Cre-cKO or D-cKO) by crossing DDX4-Cre mice with the *Hnrnpr*^flox/flox^ line. The body size, testis-to-body weight ratios and seminiferous tubule histology at P56 from D-cKO mice were comparable to Littermate *Hnrnpr*^flox/flox^ mice (Supplementary Fig. 2e–j), as well as the total epididymal sperm counts (Supplementary Fig. 3a, b). However, CASA analysis revealed strongly impaired sperm motility and an obviously increased proportion of morphologically abnormal spermatozoa in D-cKO mice (Supplementary Fig. 3c, d). Transmission electron microscopy further demonstrated structural defects in the ‘9 + 2’ axonemal microtubule arrangement, which is consistent with previous observed impaired sperm motility (Supplementary Fig. 3e–g). Concordantly, fertility testing through a 4-month period showed that D-cKO males were completely infertile (Supplementary Fig. 3h and Supplementary Table 3). Collectively, these results indicate that hnRNPR expression in germ cells is essential for proper spermatogenic process and male fertility.

### *Hnrnpr* mutations result in abnormal spermiogenesis and sperm malformation

**Fig. 3:**
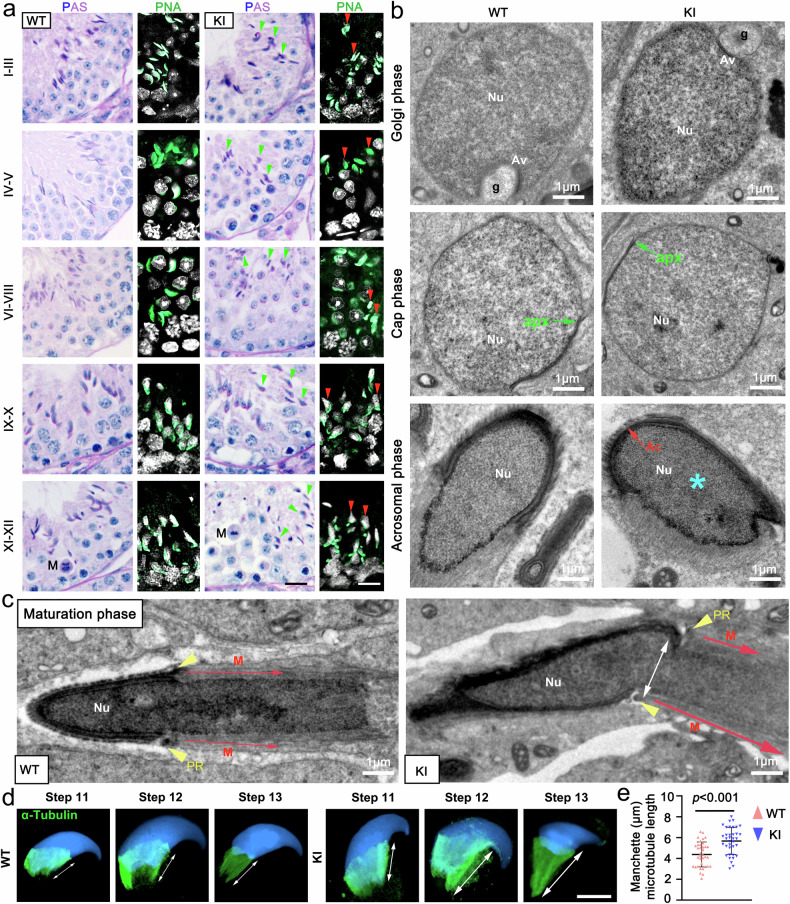
Disruption of acrosome, nuclear, and manchette assembly in KI mouse sperm. **a** PAS (left) and PNA (right) staining of seminiferous tubules from WT and KI mice at different stages of spermatogenesis. Green arrowheads (PAS) and red arrowheads (PNA) indicate aberrant acrosomal morphology in KI spermatids. M: Metaphase. Scale bars, 10 μm. **b** Transmission electron microscopy (TEM) of sperm head development in WT and KI spermatids. Nu, nucleus; g, Golgi apparatus; av, proacrosomal vesicles; apx, acroplaxome; ac, acrosome. Red arrows indicate malformed acrosomes; blue asterisks denote abnormal nuclei. Scale bars, 1 μm. **c** Representative TEM images of manchette morphology during spermatid elongation in WT and KI mice. White double-headed arrows highlight the asymmetric manchette in KI spermatids. Abbreviations: Nu, nucleus; PR, perinuclear ring; M, manchette. Scale bars, 1 μm. **d** Immunofluorescence staining of tubulin in spermatozoa from adult WT and KI mice. Tubulin marks the manchette structure; DNA was counterstained with DAPI. Scale bars, 5 μm. **e** Quantification of manchette microtubule lengths from (**d**). For each group, 40 spermatozoa were randomly selected per mouse, and average values were calculated for every 10 sperms. Data are presented as mean ± SD. *P*-values were determined using a two-sided Student’s *t*-test. *n* = 36 per group

During spermiogenesis, haploid round spermatids undergo extensive morphological remodeling and sequential, highly regulated transitions to develop into streamlined spermatozoa with the capability of motility and fertilization.^31^ To delineate the developmental defects underlying sperm malformation, we performed stage-specific luminal analysis across all 12 stages of spermatogenesis using PAS and PNA staining of testis sections (Fig. 3a). Acrosomal defects were first observed at the 9th step of spermatid development (Fig. 3a). To examine ultrastructural abnormalities, we conducted transmission electron microscopy on spermatids from various developmental stages (Fig. 3b). In WT mice, acrosome formation proceeded successfully during the Golgi and cap phases, with proper nuclear elongation and acrosome migration along the nuclear surface. In contrast, KI mice exhibited pronounced acrosomal malformations, accompanying with the developing acrosome ectopically positioned near the nucleus (Fig. 3b). Moreover, the manchette, which is a transient microtubule structure critical for shaping the sperm head and flagellum, displayed aberrant localization and excessive elongation in spermatids of KI mice (Fig. 3c). Immunostaining for α-tubulin and quantitative analysis of microtubule length confirmed that manchette structures were asymmetric and significantly longer in KI mice than in controls (Fig. 3d, e). Concurrent with these defects, nuclear elongation from KI mice was also disrupted, despite partial nuclear condensation was still evident at later stages (Fig. 3b, c). Collectively, these results demonstrate that hnRNPR is essential for proper attachment of the developing acrosome to the nuclear envelope, as well as for coordinated manchette and nuclear condensation during spermiogenesis. Therefore, loss of hnRNPR function leads to structural dysplasia and abnormal spermatozoa formation.

### The alterations of transcriptome, protein regulation and RNA splicing are attributed to *Hnrnpr* mutations

Spermiogenesis is orchestrated by a network of genes critical for male germ cells differentiation.^32,33^ To assess whether *Hnrnpr* variants affect cell populations or global gene expressions, we performed single-cell RNA sequencing (scRNA-seq) on testicular tissues from WT and KI mice (Supplementary Fig. 4a). The sequence analysis identified 22 different cell clusters in both genotypes, and cell patterns were visualized using UMAP projections (Supplementary Fig. 4b). Through bubble plots and feature maps, those clusters were further divided into seven major cell populations with distinct gene expression patterns (Supplementary Fig. 4c–e). Comparison of cell-type proportions revealed no differences between WT and KI groups (Supplementary Fig. 4f). Subclusters corresponding to spermatogonia, spermatocytes, round spermatids, and elongating spermatids were annotated, and their distributions were consistent across genotypes (Supplementary Fig. 5a, b). Gene expression signatures were further validated using violin plots and heatmaps (Supplementary Fig. 5c, d). Pseudotime trajectory analysis, cell proportion assessment, and cell cycle phase evaluation all indicated that germ cell development proceeds normally between WT and KI testes (Supplementary Fig. 5e–i).

**Fig. 4:**
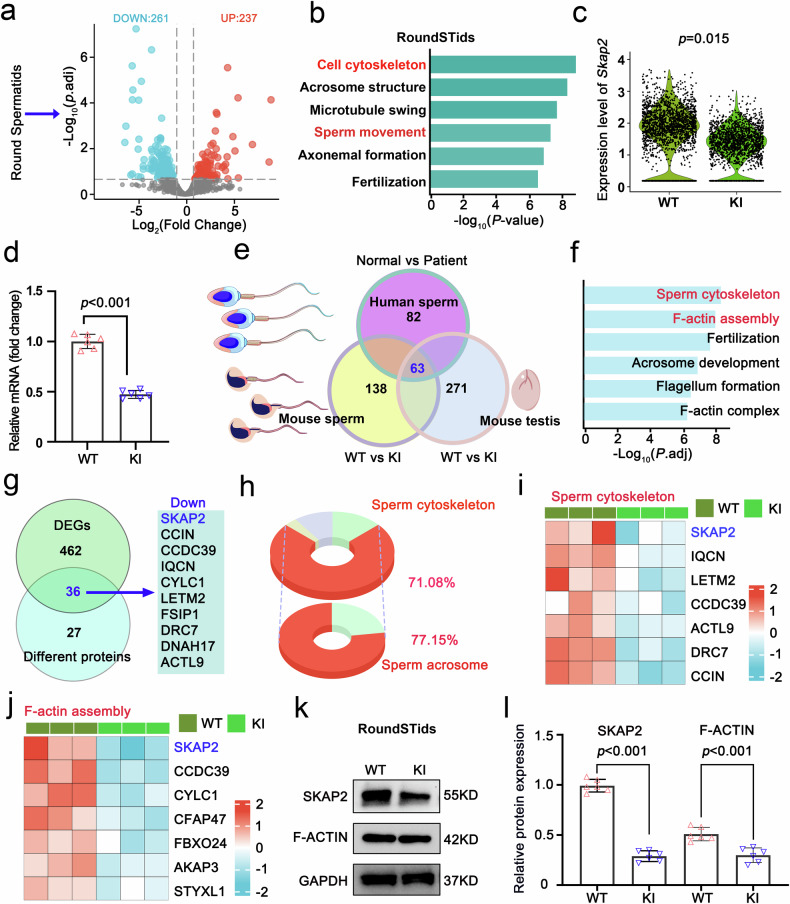
Aberrant RNA and protein profiles in KI mice. **a** Volcano plot of differentially expressed genes (DEGs) identified by single-cell RNA-seq in round spermatids from wild-type (WT) and knock-in (KI) mice at postnatal day 56 (P56). Downregulated genes are shown in blue, upregulated genes in red. **b** Gene Ontology (GO) enrichment analysis of DEGs highlights significant involvement in biological processes related to cytoskeletal organization and sperm motility. **c** Violin plots of *Skap2* mRNA expression in WT and KI round spermatids. Statistical significance was assessed using a Mann–Whitney U-test. **d** RT-qPCR validation of *Skap2*, a gene implicated in sperm cytoskeletal dynamics, confirms significantly reduced expression in purified round spermatids. Data are presented as mean ± SD; significance determined by a two-tailed Student’s *t*-test. *n* = 6 per group. **e** Venn diagram showing overlapping differentially expressed proteins identified in human sperm, mouse sperm, and mouse testis. **f** GO enrichment analysis of the overlapping proteins from (**e**) reveals significant enrichment in biological processes associated with cytoskeletal organization and F-ACTIN assembly. **g** Venn diagram illustrating the intersection between DEGs and differentially expressed proteins. **h** Doughnut chart showing that a majority (71.08%) of dysregulated proteins are associated with the sperm cytoskeleton, with a substantial fraction (77.15%) specifically linked to sperm acrosome-related structures. **i**, **j** Heatmaps depicting expression patterns of key proteins involved in cytoskeletal organization (**i**) and F-ACTIN assembly (**j**); color intensity represents relative expression levels. **k** Representative western blots showing decreased protein expression in KI round spermatids compared with WT; GAPDH served as a loading control. **l** Quantification of protein levels from (**k**) confirms significant reductions in KI versus WT. Data are presented as mean ± SD; statistical significance was determined by a two-sided Student’s *t*-test. *n* = 6 per group

Transcriptomic analysis of round spermatids of KI mice identified 498 differentially expressed genes (DEGs), including upregulated 237 genes and downregulated 261 genes (Fig. 4a). Gene Ontology (GO) enrichment highlighted corresponding processes in which cytoskeletal organization and sperm motility were listed (Fig. 4b). Among those down-regulated genes, *Skap2*, which is specifically expressed in round spermatids, showed markedly reduced mRNA expression level in the KI group, relative to WT group (Fig. 4c, d), suggesting that hnRNPR impacts sperm differentiation by regulating *Skap2* expression. Furthermore, we performed comparative proteomic analyses of testes and mature spermatozoa in both human and mouse models, identifying 63 overlapping differentially expressed proteins (Fig. 4e). GO enrichment revealed that these proteins were predominantly enriched in sperm cytoskeletal organization and F-ACTIN assembly processes (Fig. 4f). Comparative analysis of transcriptomic and proteomic datasets both revealed SKAP2 is one member of overlapping items (Fig. 4g). Of the 36 overlapping proteins, 71.08% were associated with the sperm cytoskeleton. Within this subset, 77.15% were specifically linked to acrosome formation and function (Fig. 4h). Notably, SKAP2 was highly enriched in both cytoskeletal and F-ACTIN assembly processes (Fig. 4i, j). In addition, immunoblotting confirmed significant reduction of SKAP2 and F-ACTIN protein levels in round spermatids from KI mice, compared with those from WT mice (Fig. 4k, l). Collectively, these results highlight the pivotal role of hnRNPR in spermiogenesis through modulation of SKAP2 and F-ACTIN expression, which are essential for proper cytoskeletal organization and sperm structural establishment.

Given the well-established role of hnRNP proteins in regulating alternative splicing (AS),^34^ we investigated whether hnRNPR plays the same role on AS in round spermatids (Supplementary Fig. 6a). Basing on Isoform sequencing (Iso-seq) data, we applied replicate Multivariate Analysis of Transcript Splicing (rMATS) to systematically identify AS events between WT and KI samples. The *Hnrnpr* mutation induced 1718 distinct AS events among 1197 genes (Supplementary Fig. 6b). Skipped exon (SE) events were the most prevalent, accounting for 58.73% of total AS events and 58.06% of affected genes across three biological replicates (Supplementary Fig. 6c). Read mapping analysis further revealed that *Hnrnpr* mutation significantly decreased the population of exonic reads while increasing intronic and intergenic reads, indicative of widespread splicing disruption in KI spermatids (Supplementary Fig. 6d). Gene Ontology (GO) enrichment analysis of genes impacted by aberrant AS showed strong enrichment for cytoskeleton-related activities (Supplementary Fig. 6e), which are critical for acrosome and flagellum formation during spermiogenesis. Collectively, these results demonstrate that hnRNPR is essential for maintaining normal AS patterns in round spermatids, and that its disruption leads to extensive splicing defects that may underlie the observed structural and functional abnormalities.

### hnRNPR regulates *Skap2* alternative splicing via an m6A-dependent manner

**Fig. 5:**
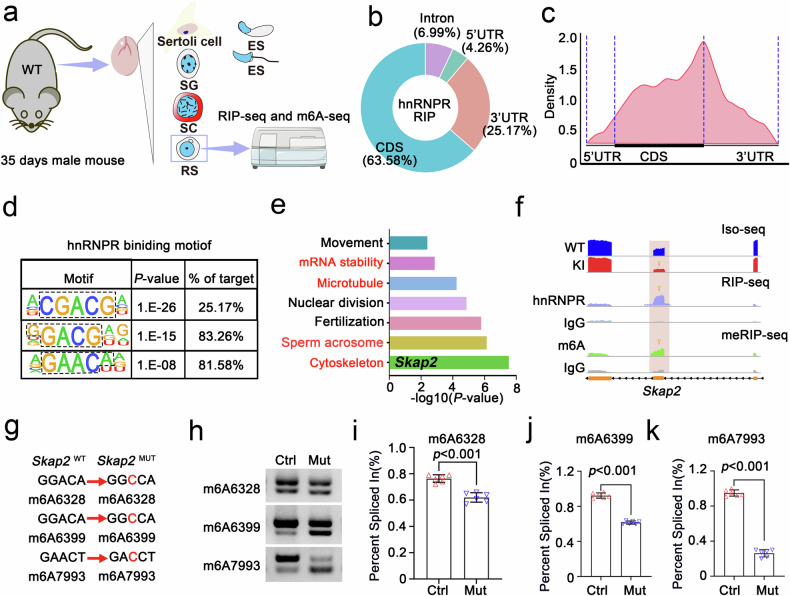
hnRNPR regulates alternative splicing of *Skap2* transcripts mediated by m6A. **a** Schematic overview of RIP-seq and m6A-seq experimental workflows. **b** Donut chart showing the genomic distribution of hnRNPR-binding peaks identified by RIP-seq in transcripts from round spermatids of P35 mouse testes. **c** Distribution of m6A peaks across transcript regions. **d** Top three enriched sequence motifs within hnRNPR-binding peaks, identified by de novo motif analysis using the HOMER algorithm; corresponding *p*-values and target percentages are indicated. **e** Gene Ontology (GO) enrichment analysis of hnRNPR-bound transcripts, highlighting significant associations with sperm cytoskeletal organization and acrosome formation. **f** IGV tracks displaying *Skap2* from Iso-seq (blue), hnRNPR RIP-seq (purple), and meRIP-seq (green). Red shading and yellow arrowheads indicate regions of alternative splicing, hnRNPR binding, and m6A modification. **g** Schematic of mutant *Skap2* minigene constructs at predicted m6A modification sites. **h**–**k** Minigene splicing assays assessing exon 2 skipping in *Skap2*. Splicing changes were detected by RT-PCR (left) and quantified as Percent Spliced In (PSI, right), representing exon 2 inclusion/exclusion levels. Data are presented as mean ± SD; statistical significance was determined using two-tailed Student’s *t*-test. *n* = 6 per group

To investigate how hnRNPR modulates pre-mRNA splicing in an m6A-dependent manner, we performed RNA immunoprecipitation (RIP) followed by high-throughput sequencing in round spermatids isolated from P35 mouse testes (Fig. 5a). RIP-seq analysis revealed 20,153 hnRNPR-binding peaks corresponding to 4687 genes from two biological replicates. Notably, these peaks were predominantly located within the coding sequences (CDS, 63.58%) and 3′ untranslated regions (3′UTRs, 25.17%) of target pre-mRNAs (Fig. 5b), which is consistent with typical m6A peak distributions (Fig. 5c). Motif analysis also indicated that hnRNPR preferentially binds CGACG-, GGACG-, and GAACA-rich sequences (Fig. 5d), typical characteristic of m6A-modified RNA. GO enrichment analysis of hnRNPR-bound transcripts highlighted significant over-representation of pathways related to cytoskeletal organization and acrosome formation, which both are important for spermiogenesis (Fig. 5e). To identify AS targets regulated by hnRNPR in an m6A-dependent manner, we integrated three datasets: hnRNPR-bound transcripts, m6A-modified RNAs, and differentially spliced genes. Our analysis revealed total 58 shared targets (Supplementary Fig. 6f). Mapping hnRNPR binding showed strong enrichment near both 5′ and 3′ splice sites, where regions are also enriched for m6A modifications (Supplementary Fig. 6g). Among these targets, *Skap2* emerged as a prominent downstream effector implicated in cytoskeletal dynamics. RIP-PCR and qPCR further confirmed direct binding of hnRNPR to *Skap2* mRNA (Supplementary Fig. 6h, i). Notably, sashimi plot analysis revealed aberrant splicing of *Skap2* in KI mice, characterized by exon 2 skipping, a defect validated by semi-quantitative RT-PCR using isoform-specific primers (Supplementary Fig. 6j, k). IGV visualization further confirmed hnRNPR binding at splice junctions shared overlap with m6A sites, implying an m6A-dependent mechanism coordinating with hnRNRP regulates the expression of *Skap2* (Fig. 5f).

To explore hnRNPR functional role in alternative splicing and its dependency on m6A, we first predicted three high-confidence m6A sites in *Skap2* using SRAMP (Supplementary Fig. 7a) and generated corresponding site-specific mutants (Fig. 5g). RIP-PCR followed by qPCR analysis revealed a significant reduction of m6A-bind *Skap2* transcripts in m6A-mutant minigene constructs (Supplementary Fig. 7b, c), confirming that the mutations could effectively disrupt m6A modification at the targeted sites. Further analyses using these site-directed mutants demonstrated that the disruption of m6A site led to decreased exon inclusion along *Skap2* gene region (Fig. 5h–k), highlighting the essential role of m6A in hnRNPR-mediated splicing regulation. Collectively, these results indicate that hnRNPR specifically modulates *Skap2* transcript splicing through an m6A-dependent mechanism, revealing post-transcriptional pathway disfunction could be a candidate for spermiogenesis defects due to *Hnrnpr* mutation.

### SKAP2 regulates sperm cytoskeletal organization to maintain spermiogenesis and male fertility

**Fig. 6:**
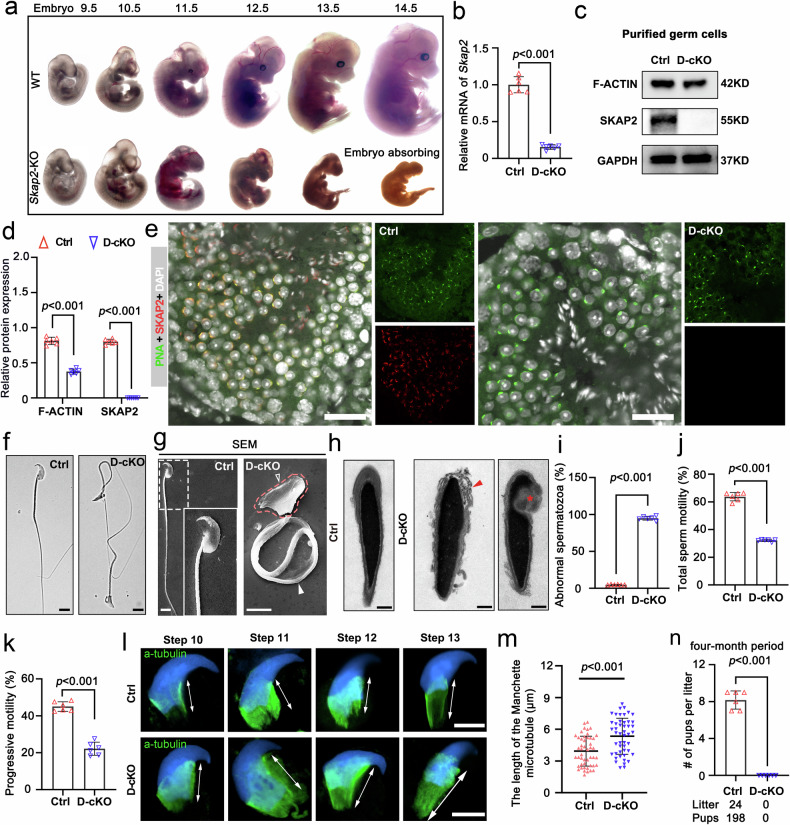
SKAP2 deficiency disrupts spermiogenesis and produces deformed spermatozoa. **a** Representative images of embryos at developmental stages E9.5-E14.5 from WT and KO mice. **b** Quantitative real-time PCR analysis of *Skap2* mRNA levels in P56 testes from control (Ctrl) and D-cKO mice. Data are presented as mean ± SD. Statistical significance was determined using a Mann–Whitney U-test. *n* = 6 per group. **c** Western blot analysis of SKAP2 and F-ACTIN protein levels in adult testes from Ctrl and D-cKO mice; GAPDH served as a loading control. **d** Densitometric quantification of protein levels in (**c**). Data are shown as mean ± SD; statistical analysis used a two-tailed Student’s *t*-test. *n* = 6 per group. **e** Representative confocal immunofluorescence images of P56 testis sections showing SKAP2 (red), PNA (sperm acrosome marker, green), and DAPI (blue) in Ctrl and D-cKO mice. Scale bars = 50 μm. **f**–**h** Representative microscopy images of spermatozoa from Ctrl and D-cKO mice: differential interference contrast (DIC) (**f**), scanning electron microscopy (SEM) (**g**), and transmission electron microscopy (TEM) (**h**). White hollow arrowhead and red dashed box indicate abnormal sperm heads; white solid arrowhead marks bent flagella; red arrowhead denotes abnormal acrosomes; red asterisk shows nuclear vacuole abnormalities. Scale bars: 5 μm (**f**, **g**), 1 μm (**h**). **i** Quantification of sperm abnormalities from 300 sperm per mouse. Data are presented as mean ± SD; statistical comparisons were made using a two-tailed Student’s *t*-test. *n* = 6 per group. **j**, **k** CASA measuring total sperm motility (**j**) and progressive motility (**k**) in epididymal sperm from Ctrl and D-cKO mice. Data are shown as mean ± SD; significance determined using a two-tailed Student’s *t*-test. *n* = 6 per group. **l** Stepwise analysis of manchette microtubule development in Ctrl and D-cKO mice. White bidirectional arrows indicate microtubule length. Scale bars = 5 μm. **m** Quantification of manchette microtubule length. For each group, 40 spermatozoa were randomly selected per mouse, and average values were calculated for every 10 sperms. Data are expressed as mean ± SD; statistical analysis used a two-tailed Student’s *t*-test. *n* = 48 per group. **n** Fertility assessment of Ctrl and D-cKO males, shown as the average number of pups per litter. Data are presented as mean ± SD; significance evaluated using a two-tailed Student’s *t*-test. *n* = 6 per group

To investigate the physiological role of SKAP2 in spermiogenesis and male fertility, we first generated a global *Skap2* knockout (*Skap2*-KO) mouse model. Homozygous *Skap2*-KO embryos, however, exhibited gestational lethality with a standstill on embryonic day 14.5 (E14.5) (Fig. 6a). This observation discolsed the critical importance of SKAP2 in early embryonic development exceeding the primitive germline. However, a loss-induced embryonic lethality also precluded direct assessment of its function in postnatal testes and adult spermatogenesis. To overcome this limitation, we generated a germline-specific conditional knockout (*Skap2* D-cKO) by crossing floxed *Skap2* mice with the DDX4-Cre line, which drives Cre recombinase expression in germ cells from embryonic day 15.5 (E15.5) onward. RT-qPCR and western blotting both demonstrated a marked reduction in *Skap2* mRNA and protein levels in D-cKO germ cells (Fig. 6b–d), confirming efficient germline-specific *Skap2* gene ablation. Immunofluorescence further verified the complete absence of SKAP2 protein in the acrosomal region of haploid spermatids in D-cKO testes (Fig. 6e), where SKAP2 is normally enriched in control mice. The establishment of complete depletion of SKAP2 in male germ cells provides an effective platform to study the role of SKAP2 in cytoskeletal organization during spermiogenesis.

Histological analysis using periodic acid-Schiff (PAS) staining revealed no overt structural abnormalities in the seminiferous tubules or epididymis, indicating that basical testicular architecture was preserved in *Skap2* D-cKO mice (Supplementary Fig. 8a–c). However, in-depth morphological examination uncovered a significant increase number of abnormal spermatozoa, particularly most of them without canonical ‘9 + 2’ axonemal microtubule arrangement in the flagellum (Fig. 6f–i and Supplementary Fig. 8d, e), revealing the loss of SKAP2 triggers ultrastructural defects in spermatozoa. CASA analysis further demonstrated a marked reduction in both total and progressive motility in D-cKO sperm compared with controls (Fig. 6j, k), phenocopying the asthenoteratozoospermia observed in *Hnrnpr* knock-in models.

Immunofluorescence staining showed pronounced reduction and erroneous localization of filamentous actin (F-ACTIN) in sperm from both KI and D-cKO mice (Supplementary Fig. 8f–i), implicating disrupted cytoskeletal dynamics and highlighting a critical role for SKAP2 in F-ACTIN assembly and spatial organization within haploid spermatids. Ultrastructural analysis also revealed aberrant manchette development, a microtubule-based structure essential for nuclear shaping and flagellum formation during spermiogenesis (Fig. 6l, m). Moreover, increased DNA fragmentation and complete failure of fertilization were observed in D-cKO mice, demonstrating that germline-specific deletion of *Skap2* leads to loss of genetic integrity and male infertility (Fig. 6n and Supplementary Fig. 8j, k and Supplementary Table 3). altogether, these findings establish SKAP2 as a pivotal regulator of cytoskeletal organization, particularly F-ACTIN dynamics, during spermiogenesis and underscore its essential role in sperm structural integrity and male reproductive competence.

### mEVs-SKAP2 ameliorates sperm motility and morphology by improving cytoskeletal remodeling and mitochondrial organization in vivo

**Fig. 7:**
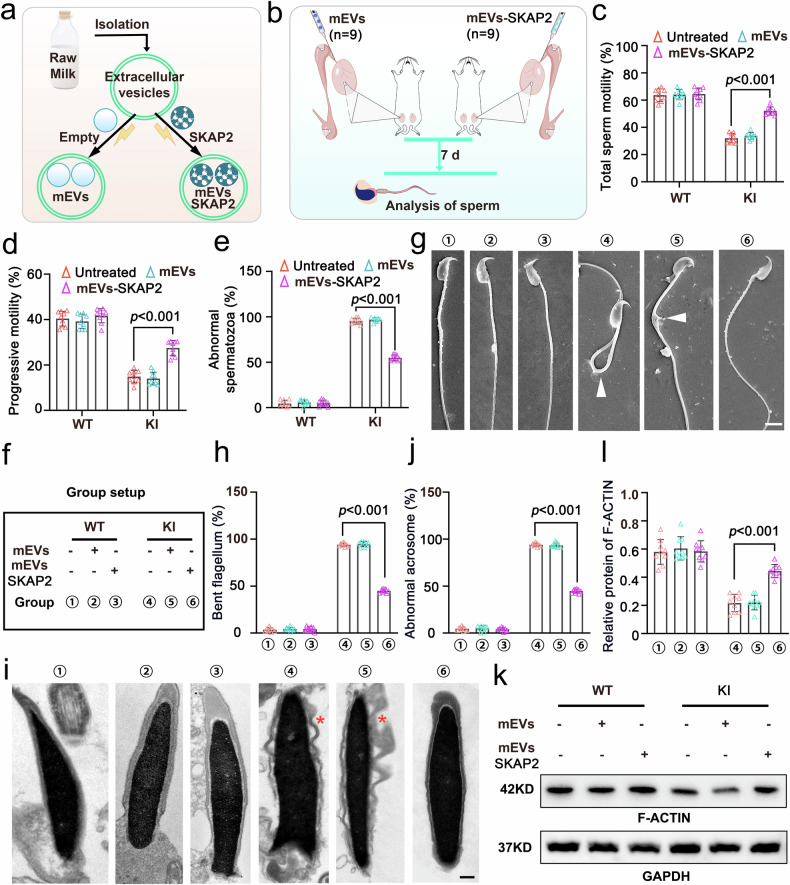
Extracellular vesicle-SKAP2 restores sperm motility and morphology in *Hnrnp*r-mutant mice. **a** Schematic of milk-derived extracellular vesicle (mEV) isolation and preparation of SKAP2-loaded mEVs (mEVs-SKAP2). “Empty” denotes EVs derived from cells transfected with a control plasmid lacking SKAP2. **b** Experimental timeline illustrating testis efferent duct injection of mEVs or mEVs-SKAP2, with sperm samples collected seven days post-injection. **c**, **d** CASA of total sperm motility (**c**) and progressive motility (**d**) in untreated, mEV-treated, and mEV-SKAP2-treated groups. Data are presented as mean ± SD; significance was determined using a two-sided Student’s *t*-test. *n* = 9 per group. **e** Quantification of morphologically abnormal spermatozoa per group (100 spermatozoa/mouse). Data are shown as mean ± SD; *p*-values calculated via two-sided Student’s *t*-test. *n* = 9 per group. **f** Group designations used for annotation in the graphs. **g** Scanning electron microscopy of sperm; white arrowhead indicates bent flagellum. Scale bars = 5 μm. **h** Quantification of sperm with bent flagella per group (100 spermatozoa/mouse). Data are mean ± SD; *p*-values by two-sided Student’s *t*-test. *n* = 9 per group. **i** Transmission electron microscopy of sperm acrosome ultrastructure; red asterisk indicates detached acrosome. Scale bars = 1 μm. **j** Quantification of abnormal acrosomes per group (100 spermatozoa/mouse). Data are mean ± SD; *P*-values calculated via two-sided Student’s *t*-test. *n* = 9 per group. **k** Representative western blot showing F-ACTIN levels in spermatozoa following in vivo injection; GAPDH served as the loading control. **l** Densitometric analysis of F-ACTIN expression from (**k**). Results are mean ± SD; significance determined by two-sided Student’s *t*-test. *n* = 9 per group

Structural and functional abnormalities in RNA-binding proteins (RBPs) are emergingly recognized as key contributors to male infertility in terms of their important effects on spermatogenesis and sperm function. Therefore, targeting RBPs and their downstream effectors could be considered as a promising therapeutic strategy. Our previous results showed that the disruption of hnRNPR, a critical RBP involved in RNA processing, could result in severely abnormal spermiogenesis. we also found the essential function of SKAP2, as a pivotal downstream effector of hnRNPR, on cytoskeletal dynamics, cell motility, signal transduction, and sperm maturation. To explore the translational potential of SKAP2 therapy, we developed SKAP2-enriched extracellular vesicles (mEVs-SKAP2), exploiting the natural cargo-delivery capabilities of EVs for targeted protein transfer (Fig. 7a). These engineered vesicles were microinjected into the efferent ducts of *Hnrnpr* knock-in (KI) mice, which exhibit sperm defects resembling human male infertility (Fig. 7b). Post-treatment analysis using CASA brought significant improvements in sperm quality: mEVs-SKAP2 treatment not only enhanced total and progressive motility, but also reduced the proportion of morphologically abnormal sperm compared with untreated and mEV-only groups (Fig. 7c–e).

Ultrastructural examination revealed a marked reduction in the occurrence of curved flagella and acrosome shedding after mEVs-SKAP2 supplement, despite the microtubular structure of the sperm axoneme remained largely unchanged (Fig. 7f–j and Supplementary Fig. 9a–d). At the molecular level, analysis of the germ cell cytoskeleton, focusing on F-ACTIN, a key component for sperm head shaping, flagellar integrity, and motility, demonstrated significant restoration in mEVs-SKAP2-treated spermatozoa, indicating partial rescue of the disrupted cytoskeletal organization caused by *Hnrnpr*-mutation (Fig. 7k, l). Moreover, replenishment of mEVs-SKAP2 into spermatogenic tubules was also beneficial for the localization of SKAP2 and F-ACTIN within spermatozoa, which could reinforce structural integrity and impel functional recovery of sperm motility and morphology (Supplementary Fig. 9e–g).

**Fig. 8:**
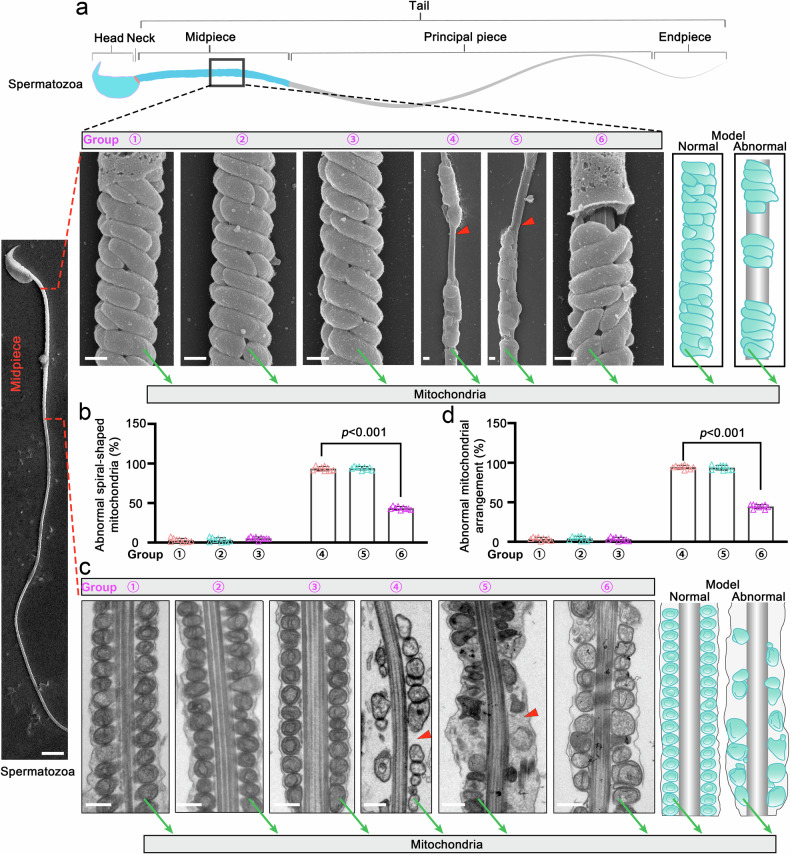
mEVs-SKAP2 restores mitochondrial organization in mouse spermatozoa. **a** Transmission electron microscopy (TEM) of sperm mid-piece mitochondria. The schematic on the right illustrates normal and abnormal mitochondrial spirals. Green arrows indicate mitochondria; red arrowheads indicate abnormal spiral-shaped mitochondria. The panel at the above and lower left shows the complete spermatozoa, with the black solid line frame and red dotted line indicating the middle section of the sperm. Scale bar: 0.3 μm in mitochondira and 5 μm in spermatozoa. **b** Quantification of abnormal spiral-shaped mitochondria from (**a**) across experimental groups. Data are shown as mean ± SD; statistical analysis was performed using two-sided Student’s *t*-test. *n* = 9 per group. **c** TEM showing mitochondrial arrangement in the sperm mid-piece. The schematic on the right depicts normal and abnormal arrangements. Green arrows indicate mitochondria; red arrowheads indicate abnormal arrangements. Scale bar: 0.5 μm. **d** Quantification of abnormal mitochondrial arrangements from (**c**) across groups. Data are shown as mean ± SD; statistical analysis via two-sided Student’s *t*-test. *n* = 9 per group

As a critical organelle, the mitochondrial sheath provides the necessary ATP to sustained sperm motility, and its defective assembly has been proved closely associated with human asthenospermia.^35^ In our study, in addition to assessing sperm morphology and the canonical ‘9 + 2’ axonemal microtubule arrangement, we also carried out a freeze-fracture method on SEM samples to obtain high resolution of mitochondrial ultrastructure within the middle segment of the sperm flagellum. Our results revealed that mEVs-SKAP2 supplementation not only restored the integrity of the mitochondrial sheath, but also enhanced its spatial organization, ultimately generating an intense ordered and compacted arrangement of mitochondria (Fig. 8a–d). These improvements are prone to support more efficient energy production and contribute to enhanced sperm motility. Taken all together, our findings highlight a previously underappreciated role of SKAP2 in regulating sperm function. Rather than rebuilding the conserved axonemal ‘9 + 2’ structure, SKAP2 appears to enhance sperm motility and morphology by promoting proper flagellar bending, stabilizing acrosome adhesion, and optimizing mitochondrial organization, thereby uncovering a novel mechanistic insight into the therapeutic potential of mEVs-SKAP2 on male infertility.

### mEVs-SKAP2 rescues sperm motility and morphology in vitro mouse and human models

**Fig. 9:**
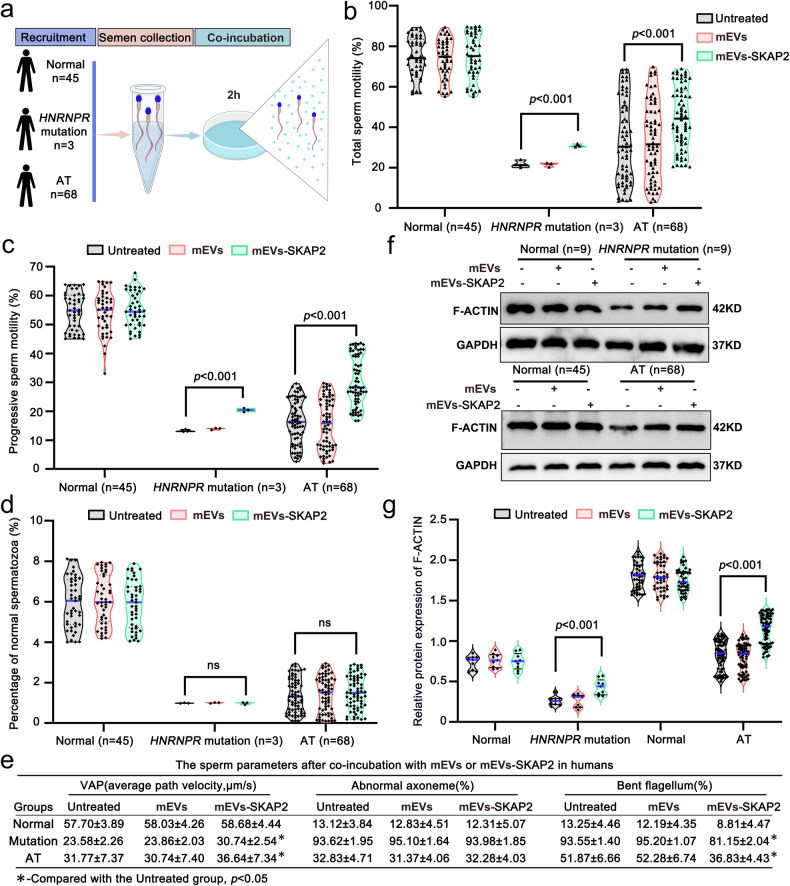
mEVs-SKAP2 ameliorates asthenoteratozoospermia in human sperm. **a** Schematic overview of the study design: volunteer recruitment, semen sample collection, and in vitro co-incubation with mEVs or mEVs-SKAP2. Post-treatment sperm samples were subjected to downstream analyses. AT, asthenoteratozoospermia. **b**, **c** CASA assessment of total (**b**) and progressive (**c**) sperm motility following co-incubation with mEVs or mEVs-SKAP2. Samples included healthy donors, patients with *HNRNPR* mutations, and individuals with idiopathic AT. Data are presented as mean ± SD; significance assessed by two-sided Student’s *t*-test. *n* = 45, 3, and 68, respectively. **d** Morphological analysis of sperm post-treatment. One hundred spermatozoa per individual were evaluated. Data are mean ± SD; statistical significance determined by two-sided Student’s *t*-test. *n* = 45, 3, and 68, respectively. **e** Evaluation of VAP (average path velocity), abnormal axoneme, and bent flagellum after co-incubation with mEVs or mEVs-SKAP2. Data are mean ± SD; significance assessed by two-sided Student’s *t*-test. *n* = 45, 3, and 68, respectively. **f** Representative western blot showing F-ACTIN protein levels in sperm following in vitro incubation. GAPDH served as the loading control. **g** Densitometric quantification of F-ACTIN expression from (**f**). Data are mean ± SD; *P*-values calculated using a two-sided Student’s *t*-test. *n* = 45, 3, and 68, respectively

To estimate the therapeutic effect of mEVs-SKAP2 on male infertility, we carried on mEVs-SKAP2 trial on human semen samples derived from three groups: normal population, individuals harboring *HNRNPR* mutations and patients diagnosed with idiopathic asthenoteratozoospermia (Fig. 9a). With the incubation of mEVs-SKAP2, we found a pronounced enhancement of sperm motility, including significantly enhanced ability of both total and progressive motility in sperm, as well as average path velocity (Fig. 9b, c, e and Supplementary Table 4). These improvements in human semen with mEVs-SKAP2 application closely mirror the functional recovery noticed in murine models (Supplementary Fig. 10a–d), underscoring the therapeutic relevance of this strategy on sperm motility between mice and human. On the contrast, mEVs-SKAP2 exhibited slight effects on sperm morphology in both humans and mice models (Fig. 9d and Supplementary Fig. 10e). Although the overall morphology of abnormal sperms remained largely unaffected with mEVs-SKAP2 co-culture, the frequency of curved flagella was significantly reduced (Fig. 9e and Supplementary Fig. 10f). Notably, this morphological improvement on sperms with mEVs-SKAP2 in vitro was less pronounced compared to those extensively rescued phenotypes observed in sperm with mEVs-SKAP2 treatment in vivo, suggesting that while sperm motility enhancement is robust across species and treatment manner, structural restoration in sperm may be more effort, relying on ex vivo conditions. Further ultrastructural analysis using transmission electron microscopy revealed that, although in vitro co-culture with mEVs-SKAP2 partially alleviated abnormal flagellar bending, the underlying axonemal organization remained largely unaltered (Fig. 9e and Supplementary Fig. 10g), which is consistent with observations in the sperms from the mice in vivo model (Supplementary Fig. 9a–d). These findings indicate that mEVs-SKAP2 predominantly exerts its effect on enhancing sperm motility by manipulating flagellar bending and mitochondrial structure rather than directly repairing axonemal ultrastructure in both human and murine sperms. Moreover, comparative analyses between in vivo and in vitro mEVs-SKAP2 applications reveals distinct advantages: direct in vivo injection yields a more robust and sustained restoration of sperm function, while in vitro co-culture, despite its relatively modest efficacy, could provide a controllable and versatile platform for mechanistic interpretation and therapeutic screening for male sperm dysfunction.

At the molecular level, treatment with mEVs-SKAP2 in the media could markedly enhance F-ACTIN polymerization compared with both untreated group and sperm exposed to mEVs lacking SKAP2 (Fig. 9f, g and Supplementary Fig. 10h, i). This cytoskeletal remodeling in sperm reflects a conserved mechanism, closely paralleling the actin reorganization observed in vivo injection model, and indicates that SKAP2 cargo within mEVs plays a pivotal role in orchestrating actin-driven structural changes. Given the crucial importance of actin dynamics in maintaining flagellar integrity and progressive motility, these findings strongly suggest that the functional recovery of sperm induced by mEVs-SKAP2 supplementation is primarily mediated through proper regulation of the actin cytoskeleton. Beyond strengthening our understanding of cytoskeletal architecture during spermiogenesis, this work also establishes a feasible therapeutic strategy for male infertility by targeting RBP-mediated signaling pathways via engineered mEVs-SKAP2 and highlights mEVs-SKAP2 as a novel, minimally invasive and highly targeted intervention with intensely translational potential for restoring sperm motility and morphology in patients with asthenoteratozoospermia.

## Discussion

Male infertility is an increasingly pressing global issue, with abnormal spermiogenesis being a major contributor to asthenoteratozoospermia. Although genetic mutations are known to disrupt spermiogenesis,^5^ their precise molecular roles in human fertilization failure remain incompletely understood. In this study, we identify *HNRNPR* as a previously unreported gene associated with asthenoteratozoospermia, providing the first evidence that homozygous mutations in an hnRNP family member can perturb human spermatogenesis. Remarkably, individuals carrying biallelic loss-of-function mutations in *HNRNPR* are viable, and this phenotype is faithfully recapitulated in both *Hnrnpr* knock-in (KI) and conditional knockout (cKO) male mouse models. Despite normal testicular architecture, these mice exhibit profound defects in sperm motility and a markedly increased frequency of morphologically abnormal spermatozoa. Together, these findings reveal a critical and evolutionarily conserved role for hnRNPR in specific stages of spermiogenesis and underscore its essential function in maintaining sperm structural and functional integrity.

RNA-binding proteins (RBPs) are central regulators of post-transcriptional processes throughout spermatogenesis,^36^ with alternative splicing serving as a key mechanism for generating transcriptomic and proteomic diversity. Nearly 95% of human protein-coding genes undergo alternative splicing, and its dysregulation contributes to approximately one-third of inherited genetic disorders,^37,38^ underscoring its biological and clinical significance. Among RBPs, hnRNPR has emerged as a multifunctional regulator that interacts with N6-methyladenosine (m6A)-modified transcripts.^17^ Deposition of m6A marks can induce local RNA structural rearrangements, known as “m6A switches,” which expose or mask binding sites for specific RBPs, thereby modulating splicing decisions and downstream genes expressions.^10^ While m6A-dependent splicing has been studied in the context of spermatogonial stem cell self-renewal^11^ and meiotic progression in spermatocytes,^16^ its role during post-meiotic spermatogenesis-particularly in the extensive cellular remodeling of round spermatids-remains largely unexplored. This stage is crucial for shaping sperm architecture and function, and misregulation can profoundly impact male fertility.^33^ Here, we reveal a previously unrecognized role for hnRNPR in mediating m6A-dependent alternative splicing of *Skap2*, a gene essential for acrosome biogenesis. These findings enhance our understanding of the spatiotemporal dynamics of m6A-mediated RNA regulation during spermiogenesis and highlight the critical role of post-meiotic alternative splicing in male germ cell differentiation and function.

Spermiogenesis involves a precisely orchestrated series of morphogenetic transitions, including acrosome formation, nuclear condensation, flagellum development, and cytoplasmic remodeling.^33^ To investigate how *Hnrnpr* mutations disrupt spermiogenesis, we performed transcriptomic profiling of round spermatids lacking functional hnRNPR. This analysis identified *Skap2* as a critical downstream effector, whose alternative splicing is markedly altered in the mutation of *Hnrnpr*. *Skap2* encodes a cytoskeletal adapter protein involved in actin remodeling and vesicle trafficking, processes essential for cellular morphogenesis and differentiation.^27^ Consistent with these roles, our data demonstrate that SKAP2 is required for proper F-ACTIN assembly during acrosome and flagellum biogenesis, pivotal steps in spermatid structural maturation. These findings expand the functional repertoire of SKAP2, establishing it as a central regulator of acrosomal development within the context of spermiogenesis. Interestingly, global *Skap2* knockout in our study resulted in embryonic lethality, in agreement with previous reports,^39^ whereas gene-trap approaches have produced viable *Skap2*-deficient mice.^40,41^ Such discrepancies likely reflect alternative splicing events that bypass gene-trap insertions, partially preserving gene function.^42^ Collectively, our results highlight the essential role of *Skap2* in both embryonic and germ cell development. Mechanistically, we establish a direct link between infertility-associated *Hnrnpr* mutations and aberrant *Skap2* splicing, revealing a previously unrecognized regulatory axis crucial for late spermatogenic progression. The delineation of this hnRNPR-SKAP2 pathway provides new insights into the molecular basis of idiopathic male infertility and identifies a potential target for future diagnostic and therapeutic strategies.

To evaluate the therapeutic potential of SKAP2, we engineered extracellular vesicles (EVs) encapsulating recombinant SKAP2 protein and assessed their ability to rescue cellular defects caused by *Hnrnpr* mutation. EVs have emerged as key mediators of intercellular communication, capable of delivering diverse bioactive molecules, including proteins, RNAs, and lipids, to recipient cells.^43,44^ In male reproduction, EVs are increasingly recognized for their roles in maintaining testicular homeostasis, modulating germ cell development, and supporting spermatogenesis.^45^ Previous studies have also shown that SKAP2 regulates F-ACTIN assembly,^25^ a process critical for normal sperm morphology and motility.^28–30,46^ Consistently, our results demonstrate that SKAP2-loaded EVs effectively restore F-ACTIN assembly and its proper localization in spermatozoa, thereby reinforcing the integrity of the cytoskeletal network. This structural recovery not only alleviates visible abnormalities, such as bent or coiled flagella, detached acrosomes, and disorganized mitochondrial sheaths-but also re-establishes the functional coupling between the cytoskeleton, mitochondria, and axonemal machinery. By stabilizing actin dynamics, SKAP2-EVs enhance flagellar mechanics, promote mitochondrial organization for efficient ATP distribution, and preserve acrosomal integrity, collectively supporting sustained motility and the acquisition of hyperactivated motility patterns. Collectively, these findings highlight SKAP2 as a promising nonhormonal therapeutic target for correcting cytoskeletal defects in late-stage sperm development and suggest that EV-based protein delivery may represent a novel strategy for treating certain forms of male infertility.

In conclusion, our study identifies hnRNPR as a key regulator of spermiogenesis in humans and mice, and a novel genetic determinant of male infertility. By integrating clinical genetics, mechanistic analyses, and therapeutic modeling, we show that hnRNPR regulates m6A-dependent alternative splicing of *Skap2*, thereby controlling acrosome formation, nuclear shaping and flagellum development. Furthermore, we provide a foundation for developing mEVs-SKAP2-based therapies aimed at restoring sperm motility and morphology through targeted modulation of sperm cytoskeletal remodeling and mitochondrial organization.

## Materials and methods

### Human sample collection

Peripheral blood samples were collected from patients with informed consent, following approval by the Institutional Ethics Committee of the Fourth Affiliated Hospital, School of Medicine, Zhejiang University (Approval No. 2024235). Candidate disease-related gene variants identified by whole-exome sequencing (WES) were validated using Sanger sequencing.^47^ Human sperm samples were obtained from infertile male patients attending the Reproductive Medicine Center of the same hospital in Yiwu. All procedures were conducted in accordance with protocols approved by the Medical Ethics Committee of the Reproductive Medicine Center and adhered to the ethical standards of the Declaration of Helsinki. Written informed consent was obtained from all participants for sample collection and publication. This study received full ethical approval under the number 2024235.

### Mice

All mouse experiments were performed using animals on a C57BL/6J inbred background. *Hnrnpr* point mutation mice were generated using the hy(e)A3A-BE4max base editor. To achieve conditional inactivation of *Hnrnpr* and *Skap2* in male germ cells, *Hnrnpr*^flox/flox^ and *Skap2*^flox/flox^ mice were crossed with DDX4-Cre mice. Genotype identification of mice using SurePlus 100 bp DNA Ladder (Hangzhou Yangming Biotechnology Co., Ltd., Amizona, AMG2001-100). Male mice at various developmental stages—from birth to adulthood—were analyzed for phenotypic characterization. All animals were housed under specific pathogen-free (SPF) conditions at 20–26 °C and 40–70% relative humidity. Veterinary care and husbandry were provided by the University Laboratory Animal Resources (ULAR) of Zhejiang University. All experimental procedures were approved by the Institutional Animal Care and Use Committee (IACUC) of Zhejiang University (Approval No.: ZJU20250714). Mice were fed and maintained by S.G.

### Fertility test in mice

A total of six WT and six KI male mice were each paired with fertile females and continuously mated over a four-month period to evaluate reproductive capacity. Throughout this time, both the number of litters produced and the number of pups per litter were carefully recorded for each pair. The cumulative reproductive output was then analyzed, and the average number of pups per litter was calculated and presented as a quantitative measure of fertility performance between groups.

### Sperm count and motility of humans and mice

Sperm count and motility were assessed via CASA. Semen samples were diluted to an appropriate concentration and loaded into a standardized counting chamber of known depth. Videos were acquired using a phase-contrast microscope equipped with a high-speed camera. CASA software automatically identified sperm heads within each field of view and tracked their trajectories across successive frames. The number of sperm detected in a given area was converted to concentration by normalizing to the chamber volume (field area × depth) and expressed as ×10⁶ sperm/mL. Multiple fields were analyzed per sample, and average values were reported.

### Criteria for abnormal sperm observation

Sperm morphology was evaluated using Diff-Quik (Funuo) and Hematoxylin and Eosin (H&E; Servicebio, G1076) staining according to the manufacturers’ protocols. Spermatozoa were classified as abnormal if they exhibited one or more morphological or structural defects. Abnormalities included head malformations (e.g., round, amorphous, detached acrosome), midpiece defects (e.g., irregular mitochondrial sheath, cytoplasmic droplet retention), and tail abnormalities (e.g., coiled, bent, or truncated flagella). At least 200 sperm per sample were examined under a phase-contrast microscope, and the percentage of abnormal sperm was calculated relative to the total number observed.

### Quantitative real-time PCR (RT-qPCR)

Total RNA was extracted from mouse samples using TRIzol reagent (Invitrogen, 15596-025) following a previously described protocol.^48^ For RNA extraction from isolated germ cells, microcellular RNA was obtained and reverse transcribed using a single-cell sequence-specific amplification kit (Vazyme, P621) according to the manufacturer’s instructions. Residual genomic DNA was removed with RNase-free DNase (Roche, 69182). Subsequently, 1 μg of total RNA was reverse transcribed into cDNA. Quantitative real-time PCR was performed using a SYBR Green master mix and gene-specific primers on a StepOnePlus Real-Time PCR System (Applied Biosystems, 4309155). Equal amounts of RNA from testes of each genotype were used for all reactions. Relative gene expression levels were calculated using the 2-ΔΔCt method, with GAPDH serving as the internal reference.

### Histology and immunofluorescence

Mouse testes and epididymides were fixed overnight at 4 °C in either Bouin’s solution (Lot# SLBJ3855V, Sigma) or 4% paraformaldehyde (PFA, Sigma, P6148) with gentle tilting and rotation. For histological analysis, tissues were dehydrated through a graded ethanol series, embedded in paraffin, and sectioned at 5 μm thickness. The staining procedure was performed using the PAS Staining Kit (Beijing Solarbio Science & Technology Co., Ltd, G1281) according to manufacturer’s protocol. Briefly, sections were oxidized with 0.5% periodic acid solution for 10 min at room temperature in the dark, then thoroughly rinsed with distilled water. Schiff’s reagent was applied for 30–60 min at 37 °C in a humidified chamber. After washing, sections were counterstained with hematoxylin for 30 s. Following dehydration through graded alcohols and xylene clearing, sections were mounted with neutral balsam. Hematoxylin and Eosin (H&E) Staining was performed using a Modified Hematoxylin-Eosin Staining Kit (Beijing Solarbio Science & Technology Co., Ltd, G1121) according to the manufacturer’s protocol. Immunofluorescence assays were performed with minor modifications to a previously described protocol. Cryosections underwent antigen retrieval and were blocked with 5% donkey serum for 1 h at room temperature (RT). Sections were incubated with primary antibodies overnight at 4 °C, followed by secondary antibody incubation for 1–2 h at RT. After washing with PBS-T Powder (1 L of 1×) from MedChemExpress (Monmouth Junction, NJ, USA; Cat# HY-K1022), sections were mounted using Vectashield mounting medium containing DAPI and imaged on a Zeiss Axio Scope.A1 microscope (Zeiss, Germany) equipped with a digital camera (MSX2, Micro-shot Technology Limited, China). Detailed information on the primary and secondary antibodies used is provided in Supplementary Table 5. We extend our gratitude to Bioss for the antibody of alpha Tubulin (bsm-33039M) and alpha Tubulin Antibody from MedChemExpress (Monmouth Junction, NJ, USA; Cat# HY-P86200). Tubulin was determined using TUBA1A Monoclonal Antibody (CSB-MA754656A0m, CUSABIO, https://www.cusabio.com/).

### Immunoblotting

Samples were dissected to remove the tunica albuginea and lysed in RIPA buffer (New Cell & Molecular Biotech, Cat# WB3100) containing a protease inhibitor cocktail in 1.5 mL Eppendorf tubes. Lysates were sonicated, centrifuged to remove debris, and mixed with 5x loading buffer (X-Blot, Xblot-0025). FuturePAGE™ 10% 12 Wells (Cat# ET12010, Boyi Biotech, China). The protein marker is provided by GenScript Corporation (Cat# MM1397-500). Proteins were then transferred onto PVDF membranes (Bio-Rad) via electroblotting. Membranes were blocked with Rapid Blocking Buffer (TBS-T) Powder (100 mL of 1×) from MedChemExpress (Monmouth Junction, NJ, USA; Cat# HY-K1027) and incubated with primary antibodies, followed by HRP-conjugated secondary antibodies for 1–2 h at room temperature. Signals were visualized using Sensitive ECL Kit from MedChemExpress (Monmouth Junction, NJ, USA; Cat# HY-K2006) and captured with the ChemiDoc XRS+ imaging system (Bio-Rad). Detailed information on all primary and secondary antibodies is provided in Supplementary Table 5. We extend our gratitude to ABclonal Technology (WuHan, China) for their antibodies of γH2AX (Cat#AP0099) and hnRNPR (Cat# A21321). SKAP2 antibody from Affinity Biosciences (Cat# DF12085). HRP Goat anti-rabbit IgG from FineTest (Cat# FNSA-0004).

### RNA immunoprecipitation (RIP)

Round spermatids were isolated from the testes of three postnatal day 35 (P35) mice and homogenized in ice-cold lysis buffer (50 mM Tris-HCl, pH 7.4; 100 mM NaCl; 0.5% NP-40; 1:100 protease inhibitor cocktail; RNase inhibitor). Following a 20-min incubation on ice to ensure complete lysis, the lysate was centrifuged at 15,000 × *g* for 10 min at 4 °C. 10% of the supernatant was reserved as the “input” sample, while the remainder was subjected to immunoprecipitation with either an anti-hnRNPR antibody or rabbit IgG as a control. RNA from both input and RIP samples was extracted using TRIzol reagent (Invitrogen). Stranded RNA-seq libraries were prepared using the KC-Digital™ Stranded mRNA Library Prep Kit for Illumina®, following the manufacturer’s instructions. This kit incorporates unique molecular identifiers (UMIs) to label cDNA molecules prior to amplification, thereby minimizing PCR and sequencing duplication bias. Libraries, with an average insert size of ~200 bp, were enriched, quantified, and sequenced on the Illumina NovaSeq 6000 platform using paired-end 150 bp reads (PE150). We extend our gratitude to Shanghai Bioprofile Technology Co., Ltd. for their contributions to RIP-seq.

### Single-cell RNA-seq sequencing and analyses

To generate single-cell suspensions, testes from three postnatal day 56 (P56) mice were first digested in RPMI 1640 containing 1 mg/mL collagenase IV (Sigma, Cat. No. C5138-100 mg) at 37 °C on a shaker for 15 min. The partially dissociated seminiferous tubules were then further digested in RPMI 1640 supplemented with 2.5 mg/mL trypsin-EDTA and 0.5 mg/mL DNase I at 37 °C for 10 min, with occasional pipetting. The resulting cell suspension was filtered through a 40 µm nylon mesh and centrifuged at 300 × *g* for 10 min at 4 °C. Cells were resuspended in PBS following the 10× Genomics protocol and loaded onto a Chromium Controller (10× Genomics) to generate single-cell cDNA libraries using the Chromium Single Cell 3′ Reagent Kits v2, according to the manufacturer’s instructions. Libraries were quantified by digital PCR and sequenced with 150 bp paired-end reads on an Illumina NovaSeq 6000 platform. Raw base call files were demultiplexed using the Cell Ranger pipeline (10× Genomics) and aligned to the mouse mm10 transcriptome. Gene expression matrices were imported into R (v4.1.2) for downstream analyses. The Seurat package was used for quality control, normalization, dimensionality reduction, clustering, and differential gene expression analysis. Quality control (QC) filtering was applied to exclude low-quality cells and potential doublets. Cells with <200 or >8000 detected genes, or with >15% mitochondrial gene content, were removed from downstream analysis. The top 2000 most variable genes were identified using the FindVariableFeatures function and used for principal component analysis (PCA) and linear dimensionality reduction. After QC, a total of 17591 cells in WT group and 17638 cells in KI group were retained for subsequent analyses. Differentially expressed genes were identified using the Wilcoxon rank-sum test, and clusters were annotated based on canonical marker genes.

### Proteomics analysis

Proteins were extracted from sperm samples and digested with trypsin. The resulting peptides were desalted using a C18 cartridge (Phenomenex) and vacuum-dried. Peptides were then reconstituted in 1% trifluoroacetic acid (TFA) and labeled with either a tandem mass tag (TMT) or an isobaric tags for relative and absolute quantitation (iTRAQ) kit. Labeled peptides were fractionated by high-pH reverse-phase high-performance liquid chromatography (HPLC) on a Thermo Betasil C18 column (5 μm, 10 mm × 250 mm) and subsequently analyzed by liquid chromatography-tandem mass spectrometry (LC-MS/MS). Raw LC-MS/MS data were processed using Proteome Discoverer (version 2.4, Thermo Scientific).

### meRIP-Seq

Total RNA was extracted from round spermatids using TRIzol reagent (15596026, Invitrogen, USA) and treated with DNase I to remove genomic DNA contamination. RNA concentration was measured with a Qubit 3.0 Fluorometer using the Qubit™ RNA Broad Range Assay Kit (Q10210, Life Technologies, USA). Polyadenylated RNA (50 μg) was enriched using VAHTS mRNA Capture Beads (N401-01/02, Vazyme, China) and fragmented into 100–200 nt fragments. Ten percent of the fragmented RNA was retained as input control, while the remainder underwent m6A immunoprecipitation. Both input and IP RNA samples were purified with TRIzol reagent. Sequencing libraries of 200–500 bp were size-selected, quantified, and sequenced on the NovaSeq 6000 platform with paired-end 150 bp reads (PE150). m6A peak calling was performed using Bedtools (v2.25.0), and enriched sequence motifs within m6A peak regions were identified using HOMER (v4.10).

### Minigenes, cell culture and cell transfection

For minigene construction, the *Skap2* genomic fragment was amplified from adult mouse testis DNA. Putative m6A methylation sites were predicted using SRAMP (Sequence-based RNA Adenosine Methylation Site Predictor),^49^ and high-confidence sites were selected for mutation (e.g., *Skap2*-Mut: A6328C, A6399C, A7993C). Both wild-type and mutant constructs were cloned into the pCDNA3.1(-) vector using the Seamless DNA Assembly Ultra Kit from MedChemExpress (Monmouth Junction, NJ, USA; Cat# HY-K1041A), and sequence-verified. HEK293T cells were cultured in DMEM (High glucose) Complete Medium (Procell, PM150210B) supplemented with 10% fetal bovine serum. Fetal bovine serum (Cat No. 209111) and cell chamber culture plate (Cat No. 725431) were procured from NEST Biotechnology Co., Ltd. (Wuxi, China). Fetal bovine serum (Cat# FBS-300) was procured from Inner Mongolia Jinyuankang Biotechnology Co., Ltd. Penicillin-Stroptomycin From Amizona Scientific LLC (Hangzhou Yangming Biotechnology Co., Ltd., Cat No. AP1001-100). The mycoplasma infection is detected using the PCR Mycoplasma Test Kit (HUABIO, Catalog# K0103, #K0104). For cell passaging, aspirate the spent medium from the cell culture dish and bottle (Bioland, CCD06-035A and CCB06-025S) and gently rinse the adherent cell layer with PBS. Transfer the cell suspension to a 15 mL centrifuge tube (Bioland, ATS05-15) and centrifuge at 1000 rpm for 3 min. Transfections were carried out using Lipo8000 according to the manufacturer’s protocol. Cells were harvested 48 h post-transfection for downstream analyses. For Cryopreservation, resuspend the cell pellet in SuperKine^TM^ Rapid Cell Cryopreservation Solution (Abbkine, Cat# BMU108). Aliquot the cell suspension into cryovials (Bioland, AC05-20) and transfer them to a −80 °C freezer for storage.

### SKAP2 protein synthesis and purification

The pGEX-6P-*Skap2* plasmid was extracted using the *SteadyPure* Plasmid DNA Extraction Kit (AG21001/AG21002, ACCURATE BIOTECHNOLOGY (HUNAN) CO.,LTD, ChangSha, China). The pGEX-6P-1 plasmid (P0005) was obtained from MiaoLingBio, China. Human or mouse *Skap2* cDNA was cloned into the pGEX-6P expression vector. Protein expression was induced by culturing the transformed cells for 12 h. Cells were harvested by centrifugation at 18,000 × *g* for 20 min, and the supernatant containing soluble SKAP2 was collected. The protein was purified sequentially using GST Nanoselector Magnetic beads (HUABIO, Catalog# 010-101-003) and GST Nanoselector Agarose (HUABIO, Catalog# 010-101-002) to achieve high purity.

### Isolation of milk-derived extracellular vesicles (mEVs)

To establish an extracellular vesicle-based SKAP2 delivery system, milk-derived extracellular vesicles (mEVs) were isolated from raw milk using a differential centrifugation protocol, as previously described. Briefly, raw milk was first centrifuged at 13,000 × *g* for 35 min to remove fat globules and cellular debris. The top fat layer and bottom pellet were discarded, and the supernatant was collected. This supernatant was then centrifuged at 100,000 × *g* for 55 min to remove large particles and microvesicles. Finally, the clarified supernatant underwent ultracentrifugation at 145,000 × *g* for 90 min to pellet the extracellular vesicles. The resulting pellet was washed three times with phosphate-buffered saline (PBS) and filtered through a 0.22 μm membrane for final purification.

### Preparation and microinjection of SKAP2-loaded mEVs

Engineered milk-derived extracellular vesicles (mEVs) were loaded with SKAP2 via electroporation using the CUY21EDIT II system (BEX, Japan). SKAP2 and mEVs were mixed at a 1:1 mass ratio in PBS, resulting in a final mEV concentration of 0.1 mg/mL. The mixture was transferred into pre-chilled 0.4 cm electroporation cuvettes and subjected to 10 electroporation cycles under the following conditions: perforation voltage 110 V, pulse duration 6 ms, interval 10 ms, secondary voltage 25 V, and capacitance 940 μF. Post-electroporation, samples were transferred to fresh tubes and incubated at 37 °C for 50 min to allow vesicle membrane recovery. The morphology and size distribution of mEVs were characterized by transmission electron microscopy (TEM) and nanoparticle tracking analysis (NTA). Protein content was quantified using the Bradford Protein Assay Kit (Cat# abs580304, absin).^50^ To assess the functional role of SKAP2 in sperm quality restoration, mEVs carrying either SKAP2 or control cargo were microinjected into the seminiferous tubules via the efferent ducts.^51^

### Statistical analysis

Unless otherwise specified in the figure legends, all data are expressed as mean ± standard deviation (SD). The Shapiro-Wilk test determines whether the data follows a Normal Distribution. The F test checks whether the variances of different sample groups are equal. When the data follows a normal distribution and has homogeneity of variance, the two-sided Student’s *t*-test should be used; when the data does not follow a normal distribution or lacks homogeneity of variance, the Mann–Whitney U test should be employed. Corresponding *P*-values are indicated in the figures and figure legends.

## Supplementary information

Supplementary Materials

## Data Availability

All data necessary to evaluate the conclusions of this study are included in the article and/or the Supplementary Materials. The datasets supporting the findings are available in the ProteomeXchange and NCBI repositories (PXD067981 and PRJNA1327596 identifiers). Additional supporting data are available from the corresponding author upon reasonable request.
