# Supplementary material for: Targeting SKAP2 restores sperm motility and morphology through modulating mitochondrial organization and cytoskeletal remodeling
Source: Signal Transduct Target Ther. 2025 Dec 24;10:416. doi: 10.1038/s41392-025-02513-3 (PMC12727759; doi:10.1038/s41392-025-02513-3)
Supplement: Supplementary file 1 — Supplementary Materials [file 41392_2025_2513_MOESM1_ESM.docx]

Supplementary Materials for

Targeting SKAP2 restores sperm motility and morphology through modulating mitochondrial organization and cytoskeletal remodeling

Shiming Gan^1, #^, Lin Yin^1, #^, Jiaming Zhou^2, #^, Sisi Li^1, #^, Shumin Zhou^3, #^, Xiaotong Yang^2^, Rui Liu^1^, Xu Fan^4^, Yangyang Li^1^, Zhendong Yao^1^, Jingshou Chen^5^, Peiran Hu^2^, Wenjing Xiong^5^, Yuan Yuan^1^, Yujiao Wen^6^, Youjiang Li^1^, Ge Jin^7^, Jianzhong Sheng^1^, Yuzhen Gao^8, *^, Hefeng Huang^1, 2, 9, *^, Chen Zhang^2, 10, *^

Correspondence to: yuzhengao@zju.edu.cn, huanghefg@zju.edu.cn, chenzhang_ired@fudan.edu.cn

**This PDF file includes:**

Materials and Methods

Figures. S1 to S10

Tables S1 to S5

Original data

Materials and Methods

Electron microscopy ultrastructural observation and analysis

For scanning electron microscopy (SEM), freshly collected sperm were were released from the epididymides of mice and smeared onto coverslips that had been pre-coated with 3-Aminopropyltriethoxysilane and fixed in electron microscopy fixative (Servicebio, G1102) for 2 hours at room temperature (RT) and subsequently stored at 4 °C for 8 hours. Samples were post-fixed in 1% OsO₄ in 0.1 M PBS (pH 7.4) for 1.5 hours at RT, dehydrated through a graded ethanol series, treated with isoamyl acetate for 15 minutes (Sinopharm, 10003128), and dried using a critical point dryer (Quorum, K850). Specimens were coated with gold particles and imaged using an S-3400N scanning electron microscope (Hitachi, Tokyo, Japan). For transmission electron microscopy (TEM), testes and sperm were minced and fixed overnight in 0.1 M cacodylate buffer (pH 7.4) containing 2.5% glutaraldehyde and 3% paraformaldehyde. After three washes in the same buffer, samples were post-fixed in 1% OsO₄ for 1 hour at 4 °C. Ultrathin sections (60-70 nm) were stained with uranyl acetate and lead citrate, then examined using a JEM-1400 transmission electron microscope (JEOL).

Freeze-fracture for SEM
To preserve native mitochondrial architecture in the sperm midpiece and expose internal mitochondrial membranes and cristae for high-resolution SEM, use rapid cryo-immobilization (high-pressure freezing or plunge-freezing), fracturing and brief freeze-etching in a dedicated freeze-fracture stage, then metal shadowing (Pt/C) and carbon backing before imaging in a cryo-SEM or by transferring replicas to a regular SEM. This approach minimizes chemical extraction and ice artifacts while creating high-contrast surface relief of internal membranes.

STA-PUT spermatogenic cell sorting
STA-PUT velocity sedimentation was used to isolate distinct germ cell populations, including spermatogonia, spermatocytes, round spermatids, and elongating spermatids. Testes from postnatal day 35 (P35) mice were enzymatically dissociated using collagenase IV (Sigma, Cat. No. C5138) and trypsin (Sigma, Cat. No. 9002-07-7) to generate a single-cell suspension. Following PBS washes, the suspension was filtered and layered onto a linear bovine serum albumin (BSA) gradient in specialized STA-PUT glassware. Germ cells were separated based on differences in sedimentation velocity, allowing collection of specific populations. Fractions achieving ≥90% purity were used for subsequent analyses.

Figure. S1.

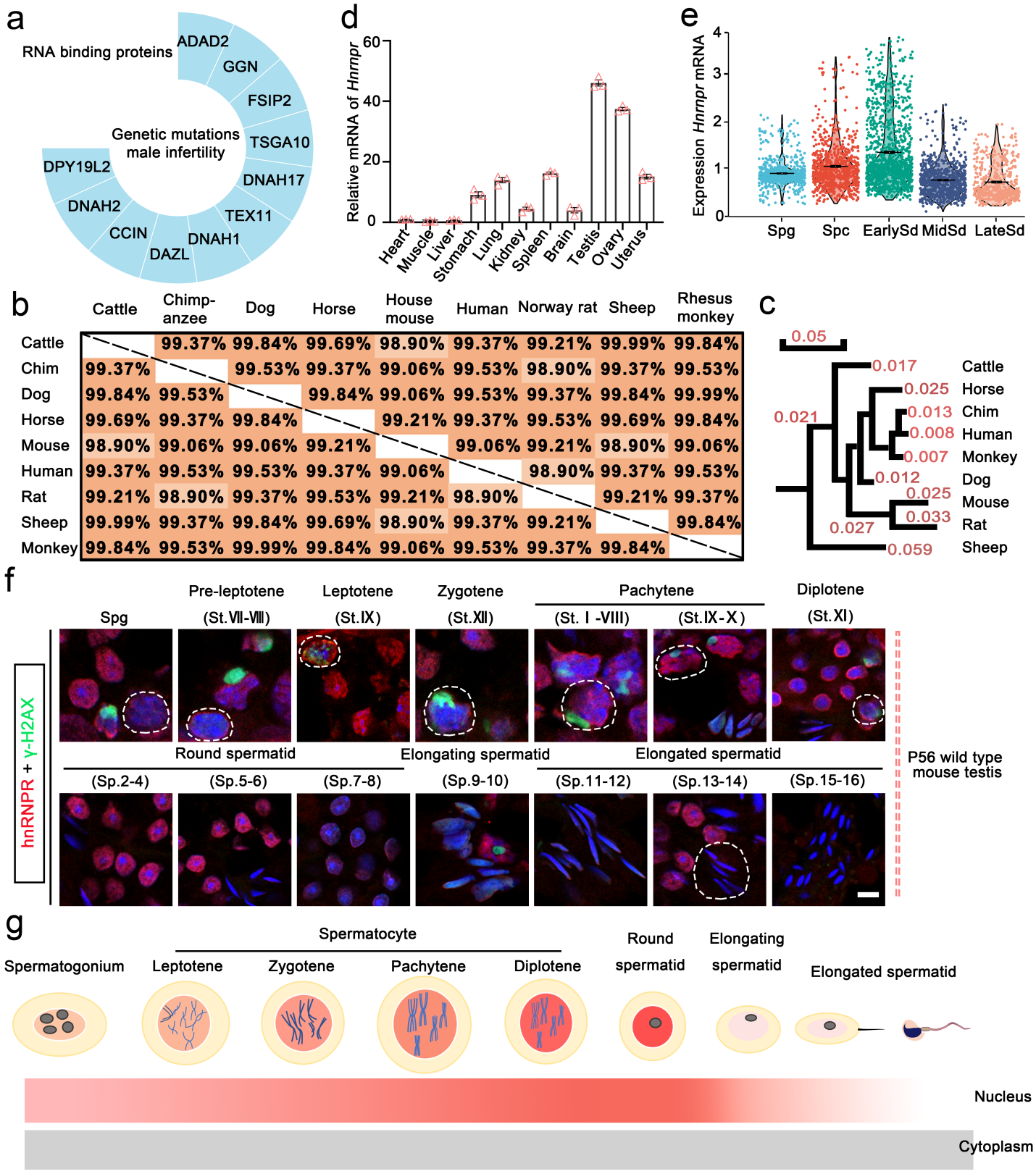


**Supplementary Fig. 1 Expression profiles of hnRNPR during mouse spermatogenesis. a** Circos plot showing 11 candidate homozygous variants of RNA-binding proteins (RBPs) related to male infertility from previous studies. **b** Multi-species sequence alignment of hnRNPR proteins showing high conservation across nine species, with 99.06% amino acid identity between human and mouse. **c** Phylogenetic analysis demonstrating evolutionary conservation of hnRNPR orthologs. **d** RT-qPCR analysis of *Hnrnpr* mRNA levels in various organs of adult wild-type (WT) mice. Data are presented as mean ± SD. n=3 per group. **e** Expression of *Hnrnpr* in distinct germ cell populations, based on single-cell RNA sequencing data (doi: 10.1016/j.celrep.2018.10.026). **f** Double immunostaining of adult WT testicular cryosections with anti-hnRNPR and anti-γH_2_AX antibodies. Dotted lines indicate specific germ cell types. Scale bars, 5 μm. **g** Temporal expression dynamics of hnRNPR during spermatogenesis in adult testes.

Figure. S2.

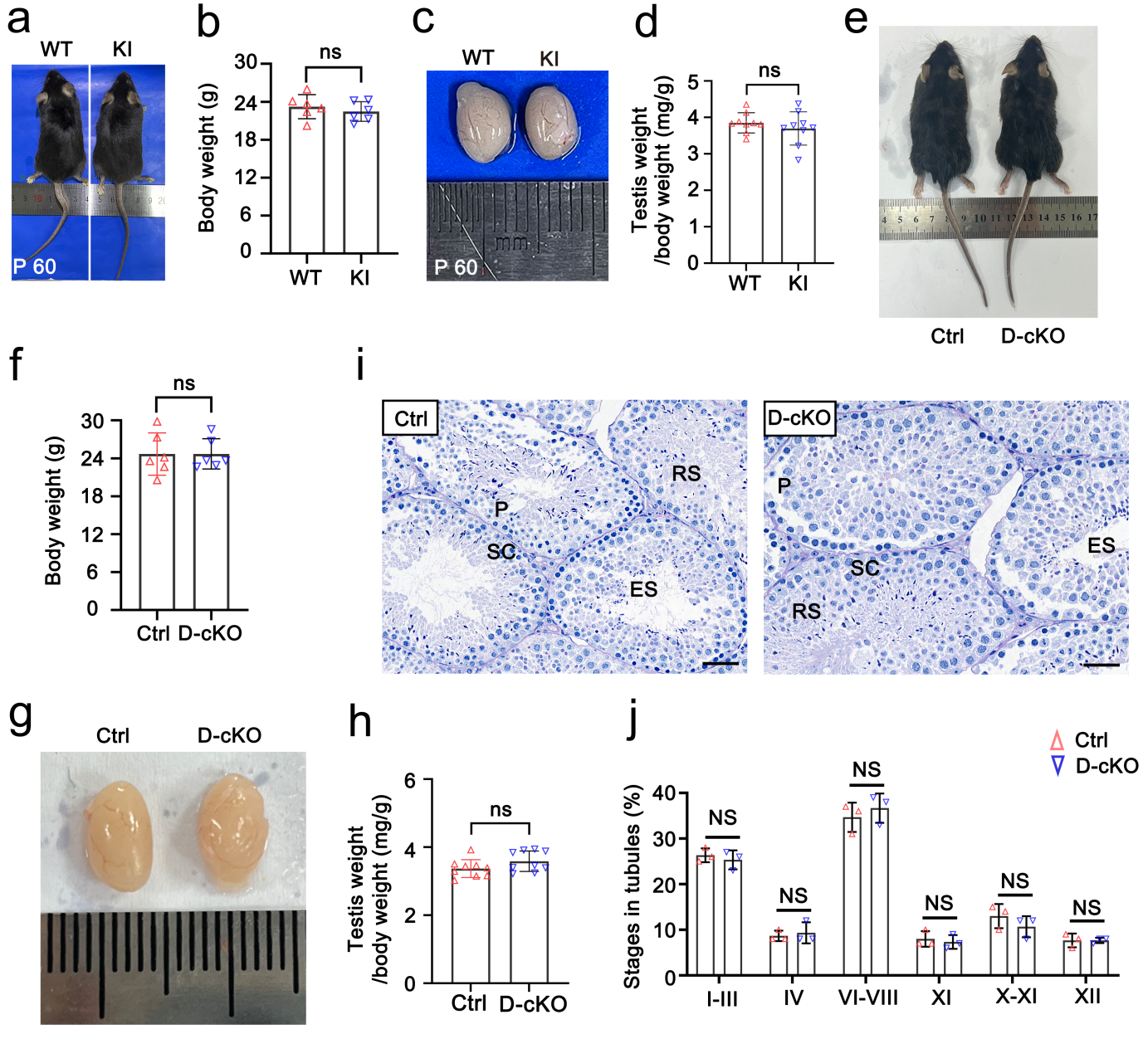


**Supplementary Fig. 2 Construction of *Hnrnpr* mutation and cKO mice. a** Gross body morphology of WT and KI mice at postnatal day 60 (P60). **b** Comparison of body weights between WT and KI mice. Data are shown as mean ± SD; *P*-values determined by two-sided Student’s *t*-test. No significant difference (ns). n=6 per group. **c** Gross morphology of testis from WT and KI mice at P60. **d** Testis-to-body weight ratio comparison between WT and KI mice. Data are presented as mean ± SD; statistical significance was assessed using a two-sided Student’s *t*-test. No significant difference (ns). n=6 per group. **e** Gross body morphology of Ctrl and D-cKO male mice at postnatal day 60 (P60). **f** Comparison of body weights between Ctrl and D-cKO mice. Data are shown as mean ± SD; *P*-values determined by two-sided Student’s *t*-test. No significant difference (ns). n=6 per group. **g** Gross morphology of testes from Ctrl and D-cKO mice at P60. **h** Testis/body weight ratio comparison between Ctrl and D-cKO mice. Data are presented as mean ± SD; statistical significance was assessed using a two-sided Student’s *t*-test. No significant difference (ns). n=6 per group. **i** Periodic acid-Schiff (PAS) staining of testes at P56. P: Pachytene spermatocyte, RS: Round spermatids, ES: Elongating and elongated spermatids, SC: Sertoli cells. Scale bars = 50 μm. **j** Quantification of abnormal seminiferous tubules, defined as lacking germ cells or specific germ cell layers, shows no significant differences between groups. Data are mean ± SD; NS, not significant. n=3 per group.

Figure. S3.

**
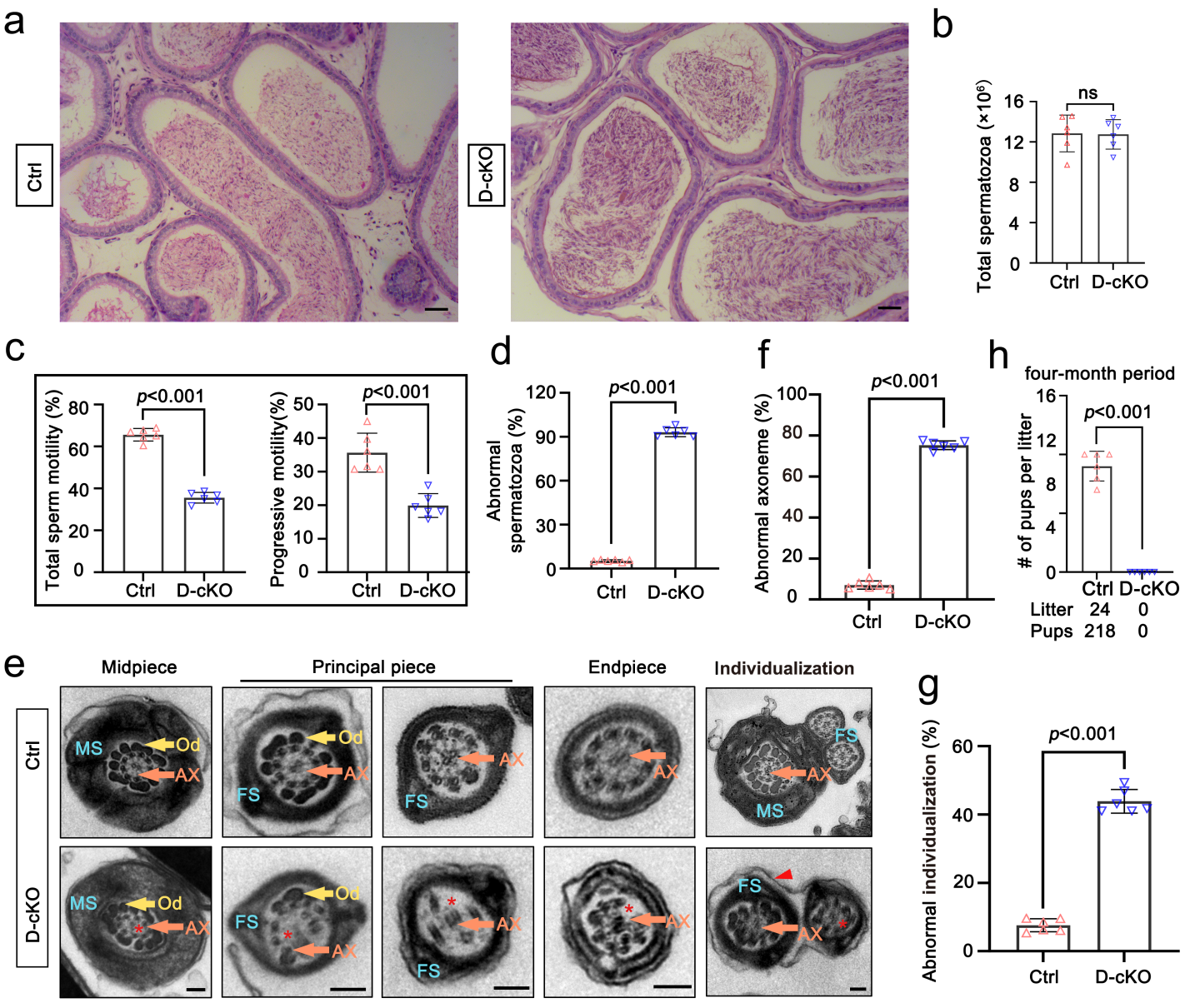

Supplementary Fig. 3 Germ cell-specific ablation of *Hnrnpr* causes sperm motility decline and male infertility. a** Representative PAS-stained cauda epididymis sections at P56. Scale bars = 25 μm. **b** Sperm counts are comparable between control and cKO mice. Data are mean ± SD; ns, not significant. n=6 per group. **c** CASA analysis reveals significant reductions in total and progressive sperm motility in cKO mice. Data are mean ± SD; two-sided Student’s *t*-test. n=6 per group. **d** Quantification of sperm morphology shows a higher proportion of abnormal sperm in cKO mice. One hundred spermatozoa were analyzed per mouse. Data are mean ± SD; two-sided Student’s *t*-test. n=6 per group. **e** Transmission electron microscopy (TEM) images reveal ultrastructural defects in the sperm flagella midpiece, principal piece, and endpiece of cKO mice. Red asterisks indicate missing axonemal microtubule doublets, and the red arrowhead highlights defective sperm individualization. Scale bars = 200 nm. Abbreviations: AX, axoneme; FS, fibrous sheath; Od, outer dense fiber; MS, mitochondrial sheath. **f, g** Quantitative analysis of axonemal and individualization defects confirms a significant increase in structural abnormalities in cKO sperm. Data are mean ± SD; two-sided Student’s *t*-test. n=6 per group. **h** Fertility assessment based on average litter size per mating shows that cKO males are infertile compared to littermate controls. Data are mean ± SD. n=6 per group.

Figure. S4.

**
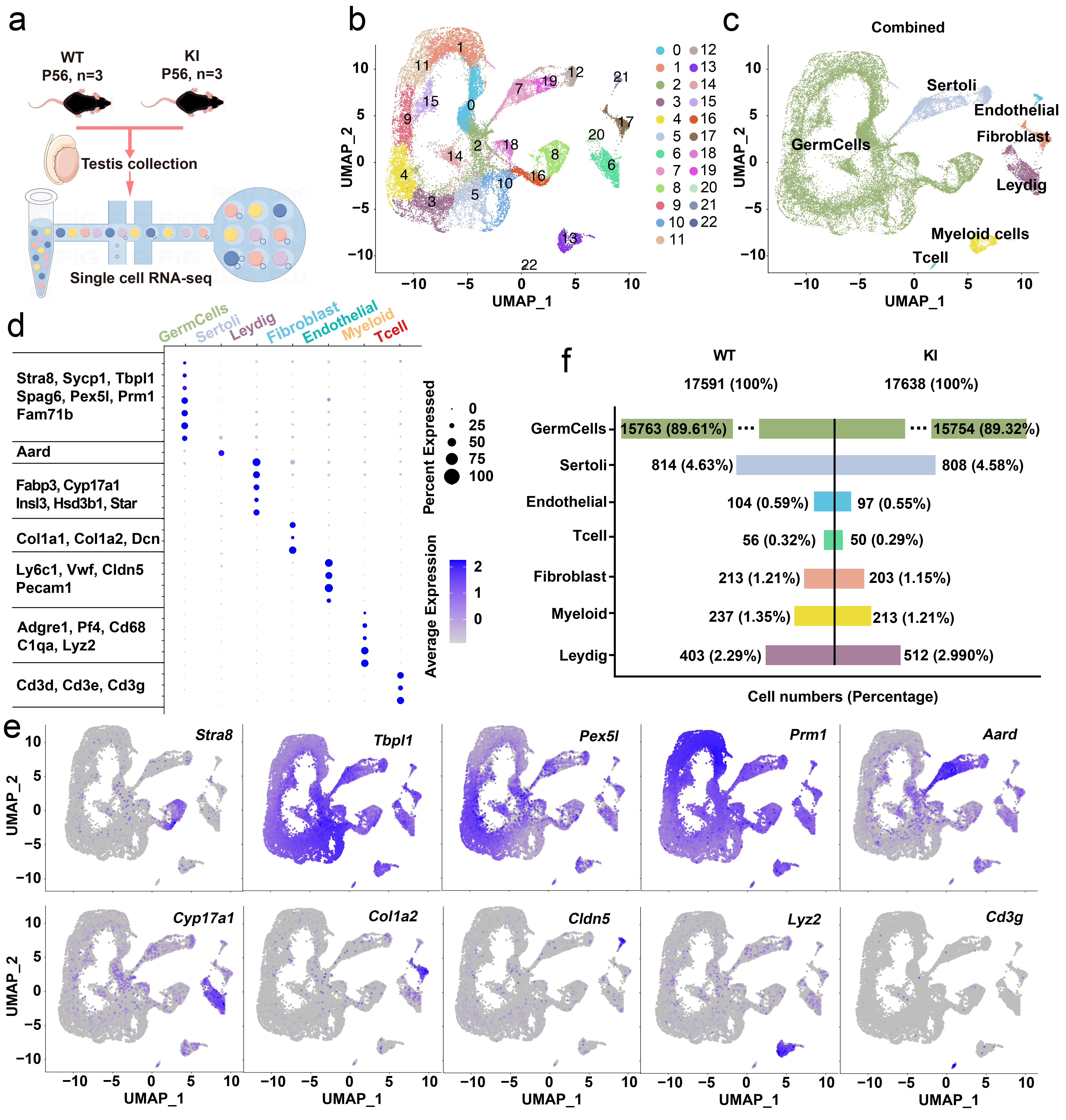

Supplementary Fig. 4 Gene expression profiling by single-cell RNA sequencing in *Hnrnpr* mutant mice at P56. a** Schematic of the experimental workflow for single-cell RNA sequencing (scRNA-seq). **b** UMAP plot showing clustering of testicular cells from WT and KI mice, revealing 22 distinct subpopulations. **c** UMAP plots highlighting seven major cell clusters in individual libraries from WT and KI testes. **d** Dot plot depicting expression levels of canonical marker genes across testicular cell types in WT and KI mice. **e** Representative expression patterns of selected genes across different cell types, with expression scales adjusted per gene. **f** Symmetrical bar plot showing relative proportions and absolute numbers of cell types in WT and KI samples. The left and right sides of the central axis indicate the percentages of each cell population in WT and KI, respectively, and the central axis has been clearly marked.

Figure. S5.

**
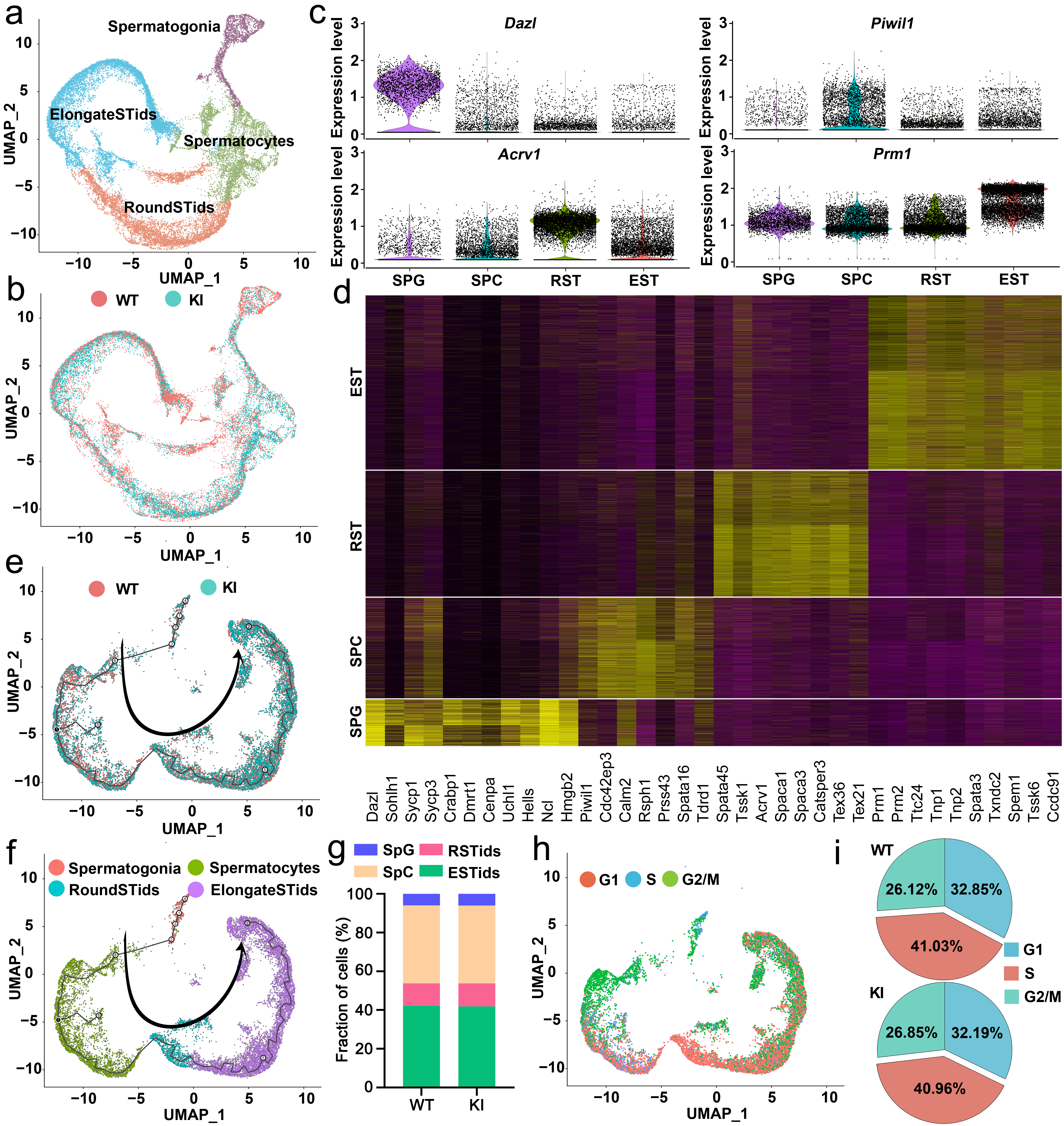

Supplementary Fig. 5 Single-cell transcriptomic analysis of germ cell populations in WT and *Hnrnpr* mutant mice. a** UMAP visualization of scRNA-seq data from germ cells in WT and KI testes, with each dot representing an individual cell and color-coded by cluster identity. **b** Combined UMAP clustering of WT and KI germ cells, with annotated clusters indicated by distinct colors. **c** Violin plots showing expression levels of marker genes across four germ cell types: spermatogonia (SPG), spermatocytes (SPC), round spermatids (RST), and elongating spermatids (EST). **d** Heatmap displaying expression profiles of identity-defining genes for each germ cell type. **e, f** Pseudotime trajectory analysis of germ cells in WT (e) and KI (f) testes, illustrating developmental progression from SPG to EST. **g** Bar plot quantifying the proportions of SPG, SPC, RST, and EST in WT and KI samples. **h** UMAP plot showing cell cycle phase assignments for germ cells from both genotypes. **i** Quantification of cells in each cell cycle phase (G1, S, G2/M) in WT and KI mice.

Figure. S6.

**
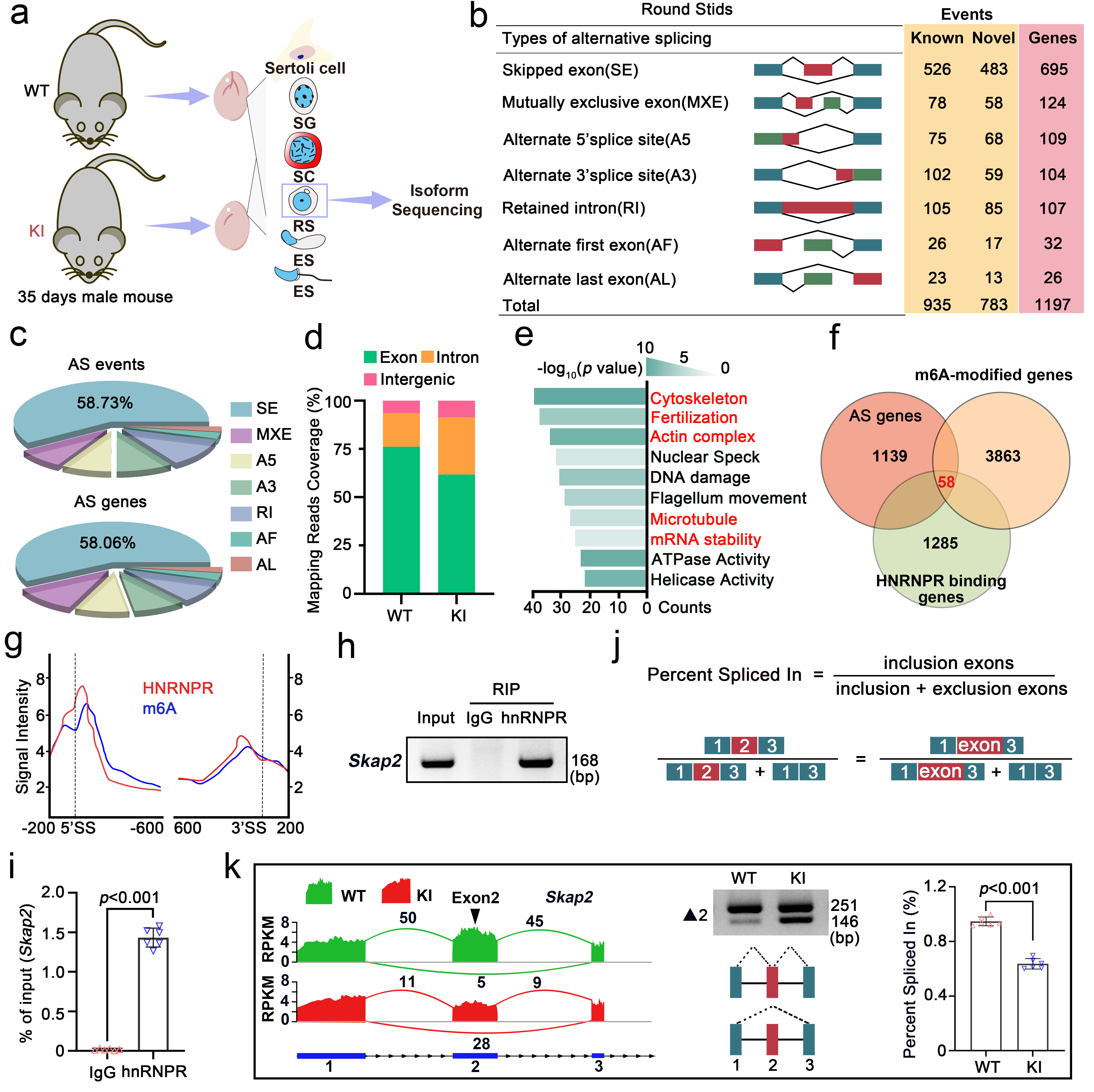

Supplementary Fig. 6 Mutation of *Hnrnpr* disrupts alternative splicing. a** Schematic of the Iso-Seq workflow and experimental design. **b** Bar plot showing the number of predicted alternative splicing (AS) events and affected genes in isolated round spermatids from P35 *Hnrnpr* KI testes compared to WT. **c** Pie charts depicting the distribution of AS event types and the corresponding genes in KI versus WT round spermatids, with different colors representing different categories. Notably, SE (skipping exon) accounts for 58.73% of splicing events and 58.06% of splicing genes. **d** Iso-Seq read coverage across exonic, intronic, and intergenic regions in WT and KI samples, indicating compromised transcriptome integrity in KI mice. **e** GO enrichment analysis of genes with significant AS alterations, highlighting roles in cytoskeletal organization and fertilization. **f** Venn diagram showing overlap among differentially spliced genes, hnRNPR RIP-seq targets, and m6A-modified transcripts, revealing shared regulatory targets. **g** Distribution of genes near splice sites (200 nt upstream and 600 nt downstream of 5′ splice sites; 600 nt upstream and 200 nt downstream of 3′ splice sites). **h** RNA immunoprecipitation (RIP) with hnRNPR antibody followed by PCR and gel electrophoresis for differentially spliced *Skap2* mRNA candidates in isolated round spermatids. **i** RIP-qPCR quantification of hnRNPR enrichment on *Skap2* mRNA in round spermatids, using IgG as a negative control. n=6 per group. Data are presented as mean ± SD; statistical significance assessed by Mann-Whitney U-test. **j** Schematic representation of alternative splicing modes. **k** Sashimi plot illustrating aberrant *Skap2* splicing in WT and KI testes based on Iso-Seq data (visualized with IGV). The right panel shows RT-PCR validation and percent spliced-in (PSI) values for *Skap2* exon skipping in P35 WT and KI round spermatids. “Δ exon number” indicates the skipped exons. Data are mean ± SD; two-tailed Student’s *t*-test. n=6 per group.

Figure. S7.

**
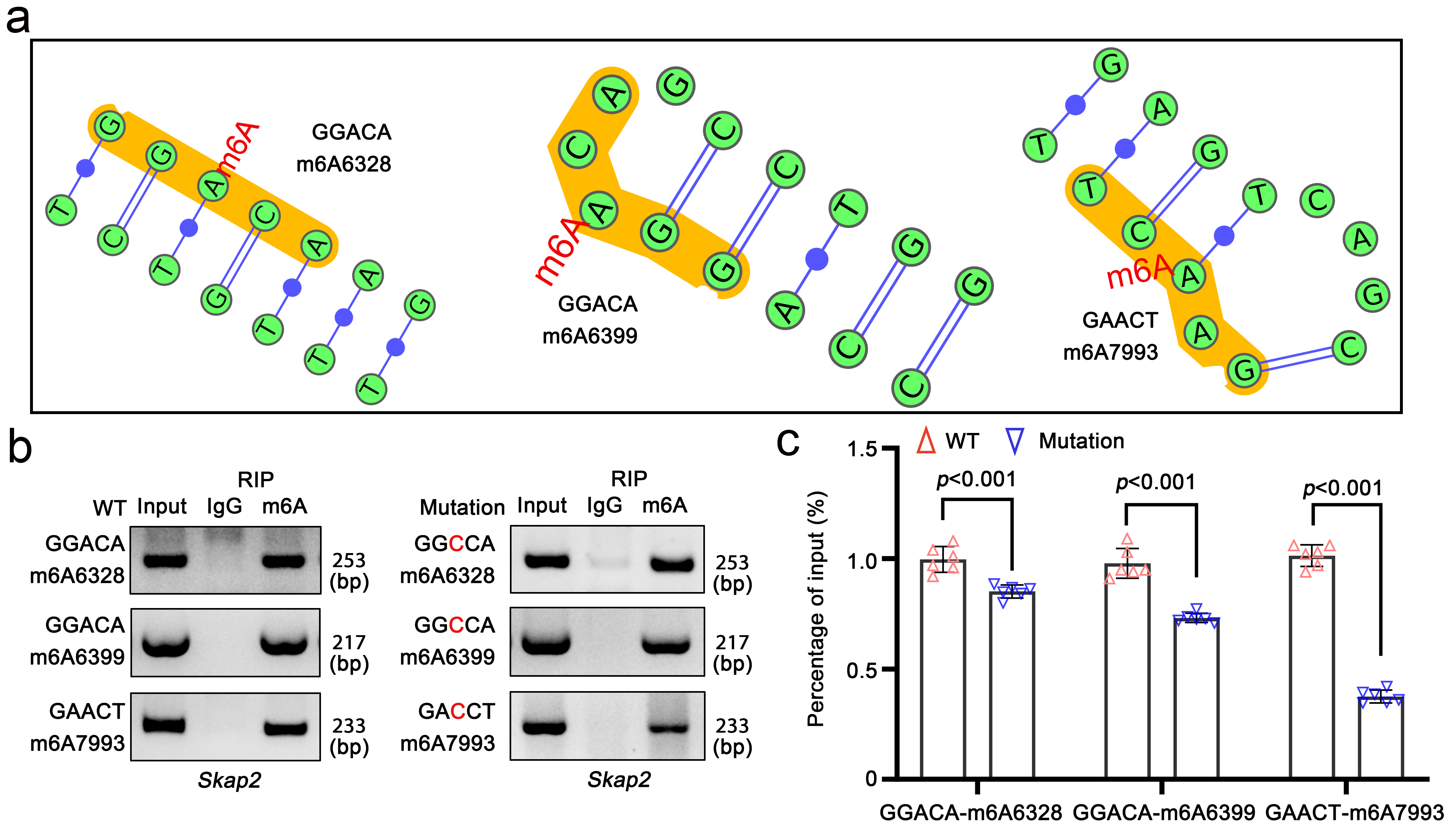

Supplementary Fig. 7 Mutation of m6A sites and validation. a** Prediction of m6A-modified sites in *Skap2* using SRAMP (Simple RNA m6A Modification Prediction). We identified a total of three highly reliable m6A sites, including the base A at position 6328, the base A at position 6399, and the base A at position 7993. **b, c** RIP-PCR (**b**) and qPCR (**c**) analyses showing preferential binding of m6A to transcripts following transfection with wild-type (WT) or m6A-mutant minigenes. RNA immunoprecipitation (RIP) was performed with anti-m6A antibodies, and anti-IgG served as a negative control. Co-precipitated *Skap2* transcripts were quantified by qPCR. n=6 per group. Data are presented as mean ± SD; statistical significance was determined using a two-sided Student’s *t*-test.

Figure. S8.

**
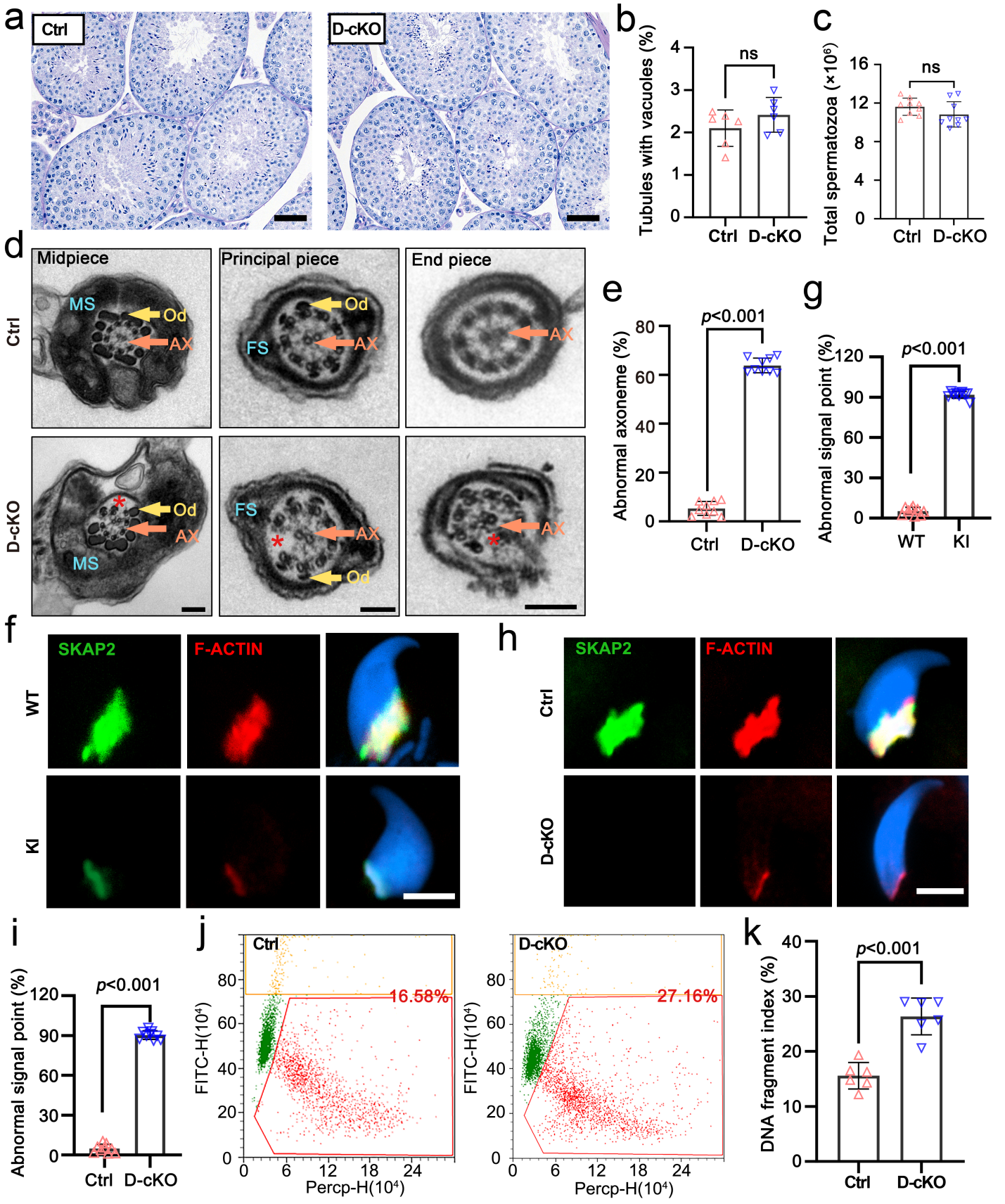

Supplementary Fig. 8 SKAP2 deficiency impairs spermiogenesis and induces sperm deformities. a** Periodic acid-Schiff (PAS) staining of seminiferous tubules in P56 *Skap2* D-cKO and littermate control testes. Scale bar, 50 μm. **b** Quantification of abnormal seminiferous tubules in Ctrl and D-cKO testes. Data are mean ± SD; ns, not significant (two-sided Student’s *t*-test). n=6 per group. **c** Quantification of total sperm counts from Ctrl and D-cKO testes. Data are mean ± SD; ns, not significant (two-sided Student’s *t*-test). n=6 per group. **d** TEM images of sperm flagella from Ctrl and D-cKO mice reveal ultrastructural abnormalities in the midpiece, principal piece, and endpiece. Labeled structures: AX (axoneme), FS (fibrous sheath), Od (outer dense fiber), MS (mitochondrial sheath). Red asterisks indicate missing axonemal microtubule doublets. Scale bars, 200 nm. **e** Quantification of axonemal abnormalities in midpiece, principal, and endpiece regions. For each mouse, 100 sections were analyzed. Data are mean ± SD (two-sided Student’s *t*-test). n=9 per group. **f** Confocal immunofluorescence images of spermatozoa from P56 WT and KI mice stained for SKAP2 (green), F-ACTIN (red), and DAPI (blue). Scale bars, 5 μm. **g** Quantification of abnormal F-ACTIN signal foci in spermatozoa. A total of 200 sperm per mouse were analyzed. n=9 per group. Data are mean ± SD (two-sided Student’s *t*-test). **h** Immunofluorescence staining for SKAP2 and F-ACTIN in spermatozoa from Ctrl and D-cKO mice. Scale bars, 5 μm. **i** Quantification of abnormal F-ACTIN signal localization in spermatozoa. A total of 200 sperm per mouse were analyzed. n=9 per group. Data are mean ± SD (two-sided Student’s *t*-test). **j** Flow cytometry analysis of sperm DNA fragmentation in Ctrl and D-cKO groups. **k** Quantification of DNA fragmentation rates showing a significant increase in D-cKO sperm. n=6 per group. Data are mean ± SD; *p*-values calculated by two-sided Student’s *t*-test.

Figure. S9.

**
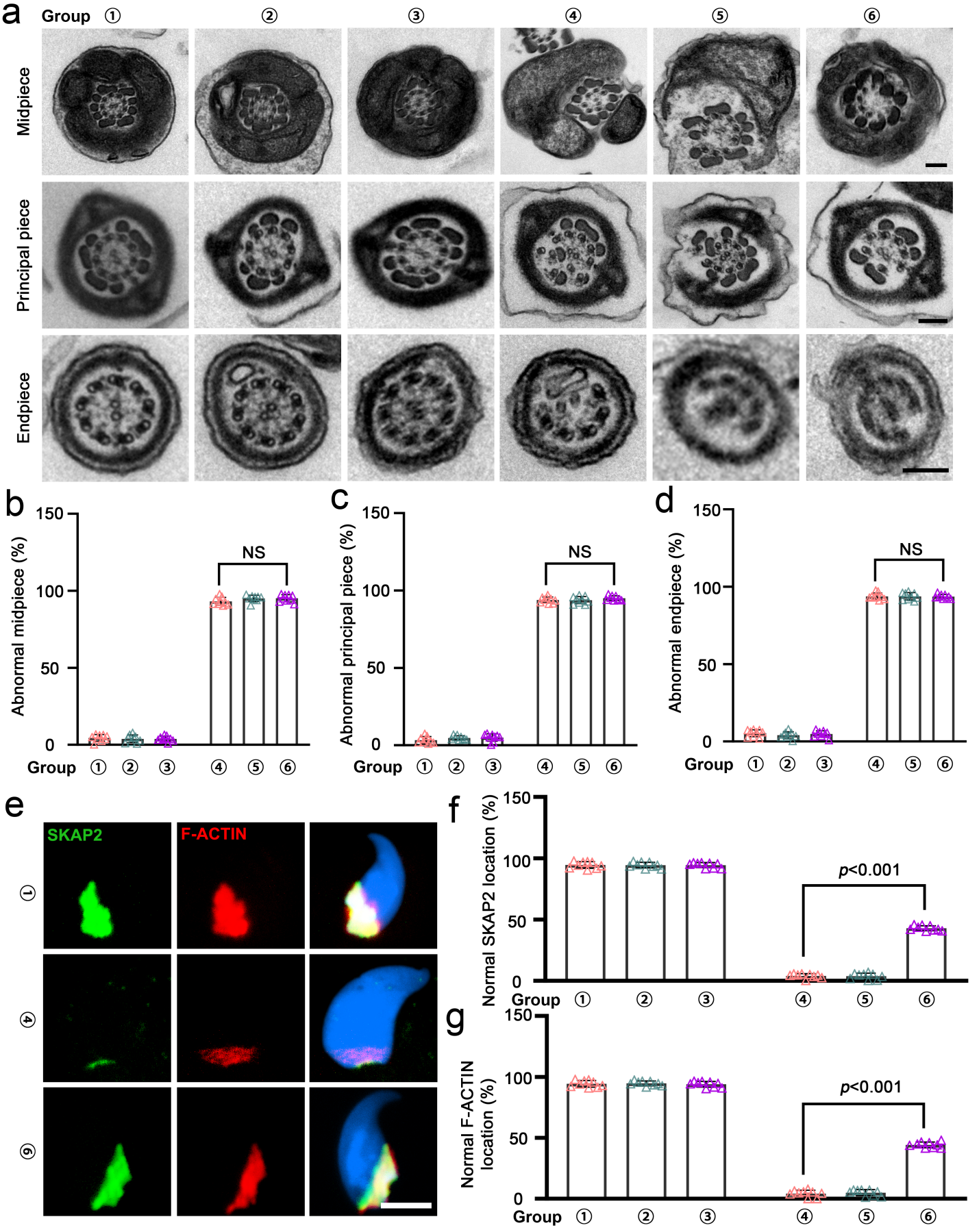

Supplementary Fig. 9 Ultrastructural changes of sperm cells following mEVs-SKAP2 injection. a** TEM images of sperm flagella in the midpiece, principal piece, and endpiece across different treatment groups. Scale bars: 200 nm. **b-d** Quantification of axonemal abnormalities in the midpiece (b), principal piece (c), and endpiece (d). For each mouse, 100 sections were analyzed. n=9 per group. Data are shown as mean ± SD; two-sided Student’s *t*-test. NS, not significant. **e** Confocal immunofluorescence images of sperm stained for SKAP2 (green), F-ACTIN (red), and DAPI (blue). Scale bars: 5 μm. **f, g** Quantification of normal SKAP2 and F-ACTIN signal foci in spermatozoa. A total of 200 sperm per mouse were evaluated. n=9 per group. Data are presented as mean ± SD; two-sided Student’s *t*-test.

Figure. S10.

**
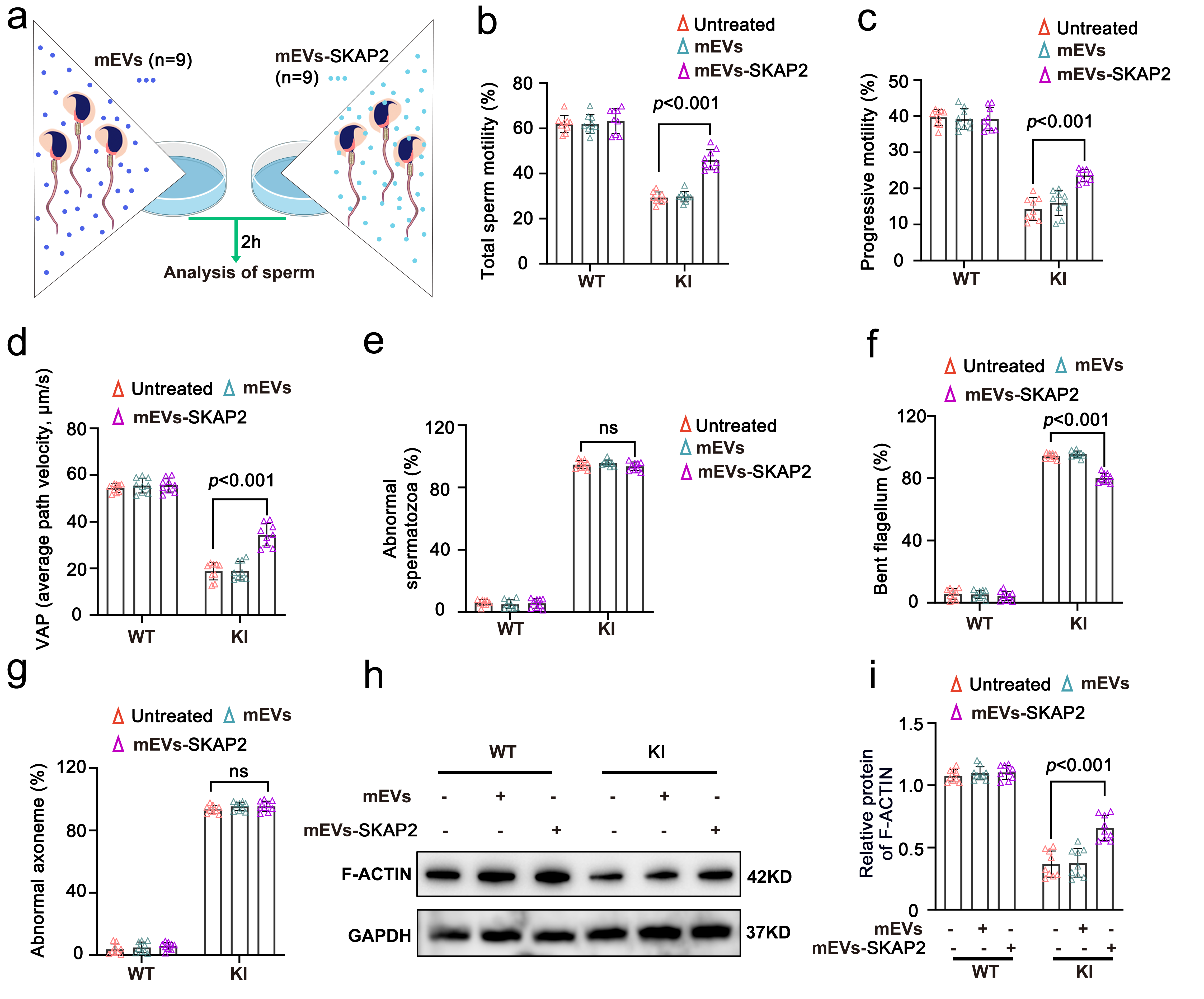

Supplementary Fig. 10 Extracellular vesicle-delivered SKAP2 restores sperm quality in vitro in *Hnrnpr*-mutation mice. a** In vitro co-incubation assay: spermatozoa were cultured with mEVs or mEVs-SKAP2 at 37 °C and 5% CO₂ for 2 h. **b-d** CASA assessment of total motility (b), progressive motility (c), and average path velocity (d) following treatment. n=9 per group. Data are mean ± SD; statistical analysis by two-sided Student’s *t*-test. **e** Histogram showing the percentage of morphologically abnormal sperm in untreated, mEVs-, and mEVs-SKAP2-treated groups. n=9 per group. Data are mean ± SD; *P* values from two-sided Student’s *t*-test; ns, not significant. **f** Quantification of flagellar bending in spermatozoa from WT and KI mice across different treatments. n=9 per group. Data are mean ± SD; two-sided Student’s *t*-test. **g** Proportion of abnormal flagellar axonemes in WT and KI sperm after treatment. n=9 per group. Data are mean ± SD; two-sided Student’s *t*-test; ns, not significant. **h** Representative western blot of F-ACTIN expression in spermatozoa following in vitro treatment, with GAPDH as a loading control. **i** Quantification of F-ACTIN expression from (h). n=9 per group. Data are mean ± SD; significance determined by two-sided Student’s *t*-test.

Table S1.

Supplementary Table 1. Clinical features of the *HNRNPR* pathogenic variants observed in three affected individuals

| **Item** | **II-1 in Family 036** | **II-2 in Family 036** | **II-1 in Family 695** |
| --- | --- | --- | --- |
| Male age | 35 | 37 | 33 |
| BMI (kg/m^2^) | 24.63 | 21.85 | 23.79 |
| Infertility years | 5 | 6 | 5 |
| Infertility | Primary infertility | Primary infertility | Primary infertility |
| Karyotype | 46, XY | 46, XY | 46, XY |
| Ultrasound | No obvious abnormality in testis | No obvious abnormality in testis | No obvious abnormality in testis |
| Habits | No smoking and drinking habits | No smoking and drinking habits | No smoking and drinking habits |
| Gene | *HNRNPR* | *HNRNPR* | *HNRNPR* |
| Transcript reference | NM_001102398.3 | NM_001102398.3 | NM_001102398.3 |
| cDNA mutation | c.1540G<A | c.1540G<A | c.1280A<C, c.1369G<A |
| Protein alteration | p.Ala514Thr | p.Ala514Thr | p.Gln427Pro, p.Gly457Arg |
| Mutation type | Missense, homozygous | Missense, homozygous | Missense, Compound Heterozygous |
| gnomAD* | 0.0002 | 0.0002 | 0.0008 |
| ExAC-EAS* | N/A | N/A | 0.0001 |
| Conservation† |  |  |  |
| Phastcons | 1 | 1 | 1 |
| Phylop | 3.052 | 3.052 | 3.077 |
| Functional prediction |  |  |  |
| SIFT | Damaging(0.001) | Damaging(0.001) | Damaging(0.001) |
| PROVEAN | Deleterious(-4.57) | Deleterious(-4.57) | Deleterious(-3.81) |
| CADD | Damaging(23.4) | Damaging(23.4) | Damaging(21.5) |
| MutationTaster | Disease causing(1) | Disease causing(1) | Disease causing(1) |
| †The Phastcons value is close to 1 when a nucleotide is conserved, and the predicted conserved sites are assigned positive scores by Phylop. *Allelic frequency of corresponding mutations in the East Asian population according to the gnomAD and ExAC browsers. | | | |

Table S2.

Supplementary Table 2. CASA analysis of semen from *HNRNPR* mutation patients.

| Item |  | Normal | c.1540G<A | p-value | Normal | c.1280A<C, c.1369G<A | p-value |
| --- | --- | --- | --- | --- | --- | --- | --- |
| **Semen Parameters** |  |  |  |  |  |  |  |
| PH | The first time | 7.5 | 7.6 |  | 7.2 | 7.5 |  |
|  | The second time | 7.2 | 7.2 |  | 7.5 | 7.3 |  |
|  | The third time | 7.3 | 7.4 |  | 7.3 | 7.4 |  |
|  | Mean | 7.3 | 7.4 |  | 7.3 | 7.4 |  |
|  | Standard deviation | 0.1 | 0.2 | 0.670 | 0.1 | 0.1 | 0.561 |
| Volum (ml) | The first time | 2.6 | 2.4 |  | 2.7 | 2.6 |  |
|  | The second time | 2.8 | 2.5 |  | 2.8 | 2.5 |  |
|  | The third time | 2.9 | 2.6 |  | 2.6 | 2.5 |  |
|  | Mean | 2.8 | 2.5 |  | 2.7 | 2.5 |  |
|  | Standard deviation | 0.1 | 0.1 | 0.065 | 0.1 | 0.0 | 0.067 |
| Concentration (10^6^/ml) | The first time | 56.3 | 60.2 |  | 68.2 | 69.5 |  |
|  | The second time | 61.2 | 58.9 |  | 76.3 | 71.3 |  |
|  | The third time | 79.5 | 71.3 |  | 58.2 | 59.6 |  |
|  | Mean | 65.7 | 63.5 |  | 67.6 | 66.8 |  |
|  | Standard deviation | 10.0 | 5.6 | 0.799 | 7.4 | 5.1 | 0.910 |
| Count (10^6^) | The first time | 126.3 | 126.7 |  | 136.5 | 142,5 |  |
|  | The second time | 132.7 | 129.5 |  | 149.2 | 149.1 |  |
|  | The third time | 142.5 | 138.6 |  | 128.6 | 133.6 |  |
|  | Mean | 133.8 | 131.6 |  | 138.1 | 141.4 |  |
|  | Standard deviation | 6.7 | 5.1 | 0.725 | 8.5 | 7.8 | 0.759 |
| **Sperm locomotion parameters** |  |  |  |  |  |  |  |
| Progressive motility, PR (%) | The first time | 50.2 | 15.9 |  | 55.6 | 14.8 |  |
|  | The second time | 48.7 | 17.1 |  | 51.2 | 15.7 |  |
|  | The third time | 53.7 | 13.5 |  | 53.8 | 16.4 |  |
|  | Mean | 50.9 | 15.5 |  | 53.5 | 15.6 |  |
|  | Standard deviation | 2.1 | 1.5 | 0.000 | 1.8 | 0.7 | 0.000 |
| Non progressive motility, NP (%) | The first time | 15.2 | 7.8 |  | 16.6 | 6.3 |  |
|  | The second time | 14.7 | 8.2 |  | 13.9 | 5.8 |  |
|  | The third time | 16.2 | 9.1 |  | 15.4 | 7.9 |  |
|  | Mean | 15.4 | 8.4 |  | 15.3 | 6.7 |  |
|  | Standard deviation | 0.6 | 0.5 | 0.000 | 1.1 | 0.9 | 0.001 |
| PR (%)+NP (%) | The first time | 65.4 | 23.7 |  | 72.2 | 21.1 |  |
|  | The second time | 63.4 | 25.3 |  | 65.1 | 21.5 |  |
|  | The third time | 69.9 | 22.6 |  | 69.2 | 24.3 |  |
|  | Mean | 66.2 | 23.9 |  | 68.8 | 22.3 |  |
|  | Standard deviation | 2.7 | 1.1 | 0.000 | 2.9 | 1.4 | 0.000 |
| **Sperm morphology parameter** |  |  |  |  |  |  |  |
| Normal sperm (%) | The first time | 5.3 | 1.1 |  | 5.5 | 1.2 |  |
|  | The second time | 5.5 | 0.8 |  | 5.3 | 0.9 |  |
|  | The third time | 5.8 | 0.9 |  | 5.7 | 0.8 |  |
|  | Mean | 5.5 | 0.9 |  | 5.5 | 1.0 |  |
|  | Standard deviation | 0.2 | 0.1 | 0.000 | 0.2 | 0.2 | 0.000 |
| A significant difference *p* <0.05 (n=3), Student’s *t*-test | | | | | | | |

Table S3.

Supplementary Table 3. Fertility test statistics.

| **Fig. 2b** | | | |
| --- | --- | --- | --- |
| ID | Litters | Average pups per litter | Total Pups |
| WT1 | 4 | 10 | 40 |
| WT2 | 5 | 9 | 45 |
| WT3 | 4 | 9 | 36 |
| WT4 | 3 | 9 | 27 |
| WT5 | 5 | 8 | 40 |
| WT6 | 4 | 7 | 28 |
| KI1 | 0 | 0 | 0 |
| KI2 | 0 | 0 | 0 |
| KI3 | 0 | 0 | 0 |
| KI4 | 0 | 0 | 0 |
| KI5 | 0 | 0 | 0 |
| KI6 | 0 | 0 | 0 |
| **Fig. 6n** | | | |
| ID | Litters | Average pups per litter | Total Pups |
| Ctrl-1 | 3 | 9 | 27 |
| Ctrl-2 | 5 | 9 | 45 |
| Ctrl-3 | 5 | 9 | 45 |
| Ctrl-4 | 4 | 8 | 32 |
| Ctrl-5 | 4 | 7 | 28 |
| Ctrl-6 | 3 | 7 | 21 |
| D-cKO-1 | 0 | 0 | 0 |
| D-cKO-2 | 0 | 0 | 0 |
| D-cKO-3 | 0 | 0 | 0 |
| D-cKO-4 | 0 | 0 | 0 |
| D-cKO-5 | 0 | 0 | 0 |
| D-cKO-6 | 0 | 0 | 0 |
| **Supplementary Fig. 3h** | | | |
| ID | Litters | Average pups per litter | Total Pups |
| Ctrl-1 | 4 | 10 | 40 |
| Ctrl-2 | 3 | 10 | 30 |
| Ctrl-3 | 5 | 10 | 50 |
| Ctrl-4 | 5 | 9 | 45 |
| Ctrl-5 | 4 | 8 | 32 |
| Ctrl-6 | 3 | 7 | 21 |
| D-cKO-1 | 0 | 0 | 0 |
| D-cKO-2 | 0 | 0 | 0 |
| D-cKO-3 | 0 | 0 | 0 |
| D-cKO-4 | 0 | 0 | 0 |
| D-cKO-5 | 0 | 0 | 0 |
| D-cKO-6 | 0 | 0 | 0 |

Table S4.

Supplementary Table 4. Analysis of semen parameters after co-incubation with mEXO or mEXO-SKAP2 in normal humans and asthenoteratozoospermia.

| Groups | VAP(average path velocity) | VAP(average path velocity)+mEXO | VAP(average path velocity)+mEXO-SKAP2 | Abnormal axoneme(%) | Abnormal axoneme(%)+mEXO | Abnormal axoneme(%)+mEXO-SKAP2 | Bent flagellum(%) | Bent flagellum(%)+mEXO | Bent flagellum(%)+mEXO-SKAP2 |
| --- | --- | --- | --- | --- | --- | --- | --- | --- | --- |
| Normal | 61.24 | 62 | 58.39 | 10.42 | 12.16 | 11.58 | 8.99 | 6.31 | 3.09 |
| Normal | 52.07 | 57.46 | 58.69 | 7.77 | 6.83 | 10.47 | 16.93 | 5.28 | 15.27 |
| Normal | 55.65 | 64.1 | 58.27 | 16.06 | 5.75 | 8.44 | 6.83 | 15.64 | 7 |
| Normal | 54.66 | 58.48 | 51.92 | 14.74 | 15.72 | 8.24 | 18.99 | 15.35 | 3.74 |
| Normal | 64.46 | 59.5 | 54.01 | 8.99 | 11.33 | 11.32 | 17.87 | 11.15 | 11.84 |
| Normal | 55.06 | 64.02 | 61.17 | 11.34 | 13.5 | 8.31 | 7.32 | 14.06 | 13.41 |
| Normal | 52 | 57.56 | 65.73 | 14.71 | 8.61 | 12.34 | 18.67 | 12.37 | 5.67 |
| Normal | 57.23 | 51.26 | 63.14 | 16.03 | 17.53 | 19.75 | 7.58 | 6.46 | 7.81 |
| Normal | 56.82 | 54.88 | 55.14 | 7.03 | 16.88 | 7.44 | 13.86 | 18.78 | 2.9 |
| Normal | 61.5 | 57.29 | 58.85 | 13.6 | 19.07 | 19.15 | 6.88 | 5.37 | 6.28 |
| Normal | 58.98 | 60.48 | 56.71 | 12.12 | 17.46 | 5.21 | 11.17 | 7.95 | 8.02 |
| Normal | 59.49 | 57.24 | 55.25 | 10.27 | 9.56 | 5.34 | 9.25 | 18.23 | 2.68 |
| Normal | 51.97 | 64.92 | 59.95 | 17.35 | 5.07 | 19.5 | 12.32 | 13.89 | 15.76 |
| Normal | 56.12 | 55.68 | 58.66 | 18.98 | 18.57 | 19.83 | 14.95 | 7.39 | 12.62 |
| Normal | 64.45 | 57.37 | 60.82 | 11.49 | 10.62 | 17 | 14.69 | 14.28 | 5.03 |
| Normal | 55.86 | 64.25 | 57.63 | 7.78 | 9.57 | 5.18 | 12.85 | 12.16 | 6.45 |
| Normal | 59.33 | 51.9 | 59.49 | 15.23 | 5.41 | 18.86 | 7.98 | 5.14 | 9.89 |
| Normal | 55.71 | 53.3 | 58.32 | 8.05 | 8.84 | 11.31 | 19.82 | 17.07 | 12.44 |
| Normal | 57.43 | 63.55 | 64.4 | 15.8 | 17.63 | 18.51 | 16.95 | 13.41 | 15.69 |
| Normal | 54.59 | 51.85 | 59.22 | 18.03 | 15.8 | 14.91 | 7.55 | 7.54 | 3.86 |
| Normal | 50.99 | 62.13 | 52.67 | 19.7 | 15.65 | 16.84 | 8.17 | 14.11 | 15.97 |
| Normal | 62.67 | 54.44 | 55.6 | 8.74 | 7.46 | 16.9 | 18.2 | 15.76 | 6.35 |
| Normal | 55.23 | 61.95 | 56.15 | 13 | 14.23 | 7.45 | 10.3 | 15.3 | 15.91 |
| Normal | 57.44 | 52.11 | 52.85 | 17.54 | 12.54 | 7.52 | 7.68 | 19.26 | 9.61 |
| Normal | 56.52 | 60.2 | 61.59 | 10.91 | 16.54 | 10.19 | 15.3 | 12.99 | 16.12 |
| Normal | 51.34 | 58.87 | 65.1 | 16.85 | 16.54 | 9.69 | 11.98 | 16.01 | 15.01 |
| Normal | 58.49 | 55.46 | 54.07 | 6.88 | 11.22 | 19.79 | 17.76 | 6.52 | 3.17 |
| Normal | 63.45 | 58.52 | 65.18 | 12.37 | 12.41 | 11.17 | 7.69 | 11.85 | 2.23 |
| Normal | 61.22 | 62.01 | 65.11 | 16.21 | 15.16 | 7.63 | 15.32 | 5.06 | 5.62 |
| Normal | 59.09 | 64.34 | 64.44 | 17.58 | 19.04 | 9.64 | 10.97 | 12.99 | 14.63 |
| Normal | 63.13 | 55.49 | 57.93 | 13.16 | 7.91 | 19.87 | 16.86 | 9.9 | 6.01 |
| Normal | 61.04 | 52.63 | 59.8 | 16.61 | 6.16 | 9.19 | 16.81 | 17.42 | 9.99 |
| Normal | 58.67 | 50.7 | 58.66 | 5.31 | 18.14 | 13.75 | 14.41 | 13.71 | 5.77 |
| Normal | 61.14 | 54.36 | 52.68 | 10.99 | 15.51 | 5.04 | 16.02 | 15.99 | 6.11 |
| Normal | 55.93 | 62.96 | 65.87 | 17.17 | 19.85 | 6.49 | 17.13 | 15.4 | 9.35 |
| Normal | 56.92 | 51.3 | 57.8 | 19.94 | 6.88 | 18.91 | 17.22 | 5.39 | 3.41 |
| Normal | 53.26 | 55.32 | 51.3 | 12.31 | 14.65 | 18.62 | 19.72 | 6.02 | 10.53 |
| Normal | 62.93 | 63.44 | 65.98 | 10.94 | 16.37 | 6.5 | 8.45 | 11.92 | 11.48 |
| Normal | 58.29 | 54.31 | 51.32 | 13.84 | 17.08 | 15.07 | 19.08 | 9.56 | 6.66 |
| Normal | 59.15 | 63.28 | 51.41 | 10.91 | 6.13 | 11.69 | 16.92 | 9.09 | 8.18 |
| Normal | 64.8 | 52.9 | 64.55 | 9.54 | 15.77 | 6.21 | 12.65 | 17.55 | 4.16 |
| Normal | 59.25 | 62.23 | 53.37 | 16.69 | 14.05 | 16.91 | 16.73 | 16.41 | 11.8 |
| Normal | 51.25 | 58.69 | 62.14 | 7.66 | 9.42 | 6.32 | 5.07 | 9.48 | 2.68 |
| Normal | 50.6 | 57.82 | 62.72 | 12.53 | 17.28 | 10.82 | 6.29 | 14.02 | 11.03 |
| Normal | 58.99 | 58.71 | 56.42 | 17.13 | 5.44 | 19.1 | 18.08 | 18.84 | 15.44 |
| HNRNPR mutation | 26.68 | 25.89 | 29.58 | 96.35 | 96.79 | 96.31 | 95.27 | 95.68 | 83.56 |
| HNRNPR mutation | 21.35 | 21.08 | 28.37 | 91.87 | 92.88 | 93.85 | 91.83 | 93.72 | 81.32 |
| HNRNPR mutation | 22.7 | 24.61 | 34.26 | 92.65 | 95.62 | 91.79 | 93.55 | 96.21 | 78.56 |
| AT | 37.15 | 40.36 | 25.73 | 30.06 | 37.78 | 27.48 | 9.21 | 8.28 | 19.03 |
| AT | 27.28 | 19.41 | 29.95 | 36.38 | 38.69 | 25.43 | 51.8 | 59.97 | 42.11 |
| AT | 37.39 | 34.45 | 27.02 | 39.67 | 27.05 | 29.56 | 49.76 | 52.45 | 35.21 |
| AT | 22.86 | 23.38 | 41.31 | 31.87 | 29.84 | 31.3 | 48.36 | 55.01 | 44.23 |
| AT | 42.37 | 19.37 | 33.61 | 36.02 | 30.8 | 31.63 | 53.28 | 57.51 | 39.37 |
| AT | 27.71 | 27.6 | 41.35 | 37.4 | 26.13 | 32.44 | 46.08 | 57.3 | 37.75 |
| AT | 26.8 | 29.69 | 27.51 | 38.33 | 29.86 | 37.24 | 59.32 | 46.78 | 40.43 |
| AT | 20.19 | 20.6 | 40.36 | 28.2 | 32.07 | 34.04 | 55.73 | 49.25 | 37.46 |
| AT | 26.65 | 39.62 | 44.98 | 26.86 | 27.03 | 32.49 | 54.23 | 47.17 | 36.18 |
| AT | 26.99 | 34.87 | 38.38 | 28.07 | 37.51 | 32.32 | 58.37 | 46.96 | 36.09 |
| AT | 26.83 | 25.34 | 42.05 | 36.27 | 30.03 | 30.04 | 51.37 | 57.94 | 40.97 |
| AT | 35.56 | 35.78 | 34.85 | 36.24 | 28.06 | 34.89 | 48.72 | 57.11 | 38.07 |
| AT | 41.9 | 37.38 | 49.7 | 32.24 | 37.66 | 27.52 | 59.56 | 55.6 | 34.33 |
| AT | 33.5 | 23.38 | 35.94 | 27.06 | 34.43 | 39.65 | 50.47 | 56.68 | 35.71 |
| AT | 31.46 | 39.67 | 45.19 | 34.88 | 37.4 | 32.32 | 51.75 | 48.72 | 34.13 |
| AT | 31.89 | 27.88 | 31.37 | 28.99 | 28.51 | 29.22 | 45.91 | 59.46 | 34.76 |
| AT | 43.8 | 30.87 | 49.28 | 26.21 | 29.12 | 38.53 | 50.93 | 47.02 | 41.45 |
| AT | 40.16 | 28.26 | 31.81 | 38.31 | 39.02 | 27.58 | 46.86 | 50.29 | 41.91 |
| AT | 32.43 | 38.69 | 45.42 | 39.3 | 26.59 | 30.76 | 53.63 | 55.99 | 36.56 |
| AT | 29.51 | 29.15 | 47.01 | 39.23 | 34.27 | 36.53 | 58.43 | 55.07 | 40.08 |
| AT | 29.57 | 19.98 | 26.68 | 39.79 | 26.31 | 26.46 | 51.03 | 59.9 | 32.91 |
| AT | 23.9 | 42.8 | 42.41 | 25.67 | 30.02 | 35.58 | 50.87 | 47.54 | 40.47 |
| AT | 32.6 | 31.2 | 25.55 | 37.95 | 27.66 | 36.9 | 47.6 | 48.67 | 37.12 |
| AT | 26.64 | 28.59 | 30.47 | 30.84 | 34.11 | 35.68 | 52.89 | 53.33 | 36.7 |
| AT | 42.91 | 36.87 | 42.3 | 28.19 | 35.19 | 31.67 | 50.97 | 50.24 | 32.42 |
| AT | 40.9 | 32.89 | 40.76 | 25.98 | 34.52 | 30.78 | 57.62 | 55.55 | 39.68 |
| AT | 25.51 | 43.34 | 32.57 | 39.37 | 27.6 | 32.45 | 45.1 | 53.45 | 33.02 |
| AT | 42.02 | 36.53 | 37.38 | 26.36 | 33.45 | 25.64 | 53.59 | 54.77 | 33.39 |
| AT | 21.89 | 40.04 | 44.85 | 37.82 | 31.83 | 36.29 | 48.59 | 56.48 | 31.66 |
| AT | 42.65 | 30.14 | 39.21 | 33.86 | 33.3 | 26.56 | 57.66 | 52.85 | 44.78 |
| AT | 23.19 | 21.07 | 49.91 | 38.78 | 27.34 | 27.23 | 50.06 | 49.07 | 33.99 |
| AT | 37.7 | 41.47 | 27.52 | 37.69 | 29.13 | 28.75 | 53.29 | 46.22 | 36.14 |
| AT | 37.82 | 19.54 | 25.86 | 29.95 | 29.77 | 28.32 | 58.1 | 53.98 | 43.3 |
| AT | 19.52 | 42.29 | 33.34 | 27.41 | 25.09 | 39.9 | 57.83 | 51.4 | 32.81 |
| AT | 19.81 | 23.18 | 49.94 | 35.86 | 33.78 | 29.15 | 53.02 | 58.47 | 33.26 |
| AT | 21.58 | 27.85 | 26.12 | 31.99 | 36.5 | 32.27 | 50.43 | 48.17 | 41.93 |
| AT | 32.28 | 19.67 | 34.23 | 31.56 | 35.15 | 32.02 | 57.16 | 56.63 | 34.44 |
| AT | 35.58 | 23.11 | 44.28 | 32 | 34.45 | 28.38 | 45.19 | 55.42 | 33.33 |
| AT | 38.77 | 21.51 | 46.86 | 34.55 | 25.19 | 31.75 | 56.65 | 45.78 | 42.07 |
| AT | 39.38 | 43.23 | 45.34 | 32.84 | 26.62 | 25.85 | 49.08 | 59.48 | 40.07 |
| AT | 33.88 | 21.29 | 45.01 | 30.76 | 33.89 | 39.18 | 59.04 | 45.46 | 41.22 |
| AT | 26.68 | 27.52 | 28.11 | 27.1 | 26.63 | 31.76 | 47.63 | 47.4 | 31.26 |
| AT | 32.81 | 31.4 | 30.22 | 39.47 | 33.89 | 29.37 | 58.08 | 52.41 | 32.92 |
| AT | 21.08 | 30.59 | 38.4 | 28.74 | 39.24 | 35.7 | 48.52 | 48.61 | 37.36 |
| AT | 30.45 | 38.77 | 35.28 | 25.35 | 25.47 | 29.46 | 58.1 | 52.97 | 34.04 |
| AT | 22.38 | 29.69 | 28.23 | 39.69 | 28.8 | 25.26 | 54.62 | 54.95 | 31.13 |
| AT | 21.06 | 39.28 | 31.53 | 38.14 | 30 | 32.63 | 46.67 | 58.24 | 40.86 |
| AT | 23.24 | 28.84 | 28.76 | 36.95 | 29.22 | 36.92 | 52.72 | 50.75 | 43.94 |
| AT | 35.05 | 31.96 | 34.07 | 27.74 | 25.15 | 37.16 | 45.41 | 53.84 | 31.33 |
| AT | 39.08 | 19.08 | 25.44 | 39.91 | 39.14 | 32.65 | 50.83 | 55.73 | 40.98 |
| AT | 41.97 | 36.47 | 49.55 | 39.56 | 35.13 | 34.81 | 47.65 | 51.05 | 39.88 |
| AT | 37.18 | 19.86 | 36.46 | 36.94 | 31.86 | 34.2 | 52.47 | 52.22 | 31.05 |
| AT | 43.45 | 31.85 | 37.85 | 38.4 | 31.18 | 34.09 | 52.59 | 51.03 | 42.16 |
| AT | 34.23 | 36.57 | 40.91 | 26.28 | 30.18 | 29.18 | 57.48 | 53.95 | 32.77 |
| AT | 39.98 | 38.31 | 31.51 | 26.42 | 34.8 | 33.9 | 54.69 | 53.97 | 37.1 |
| AT | 37.38 | 36.66 | 36.58 | 30.76 | 29.95 | 37.58 | 56 | 52.12 | 38.6 |
| AT | 43.35 | 28.04 | 30.55 | 33.14 | 28.21 | 36.64 | 50.45 | 54.72 | 35.28 |
| AT | 27.12 | 23 | 44.67 | 33.65 | 27.29 | 31.65 | 52.54 | 55.5 | 35.48 |
| AT | 36.93 | 28.43 | 43.47 | 29.63 | 33.21 | 25.66 | 58.75 | 59.09 | 42.34 |
| AT | 27.69 | 20.48 | 37.74 | 29.54 | 36.15 | 37.32 | 58.45 | 47.78 | 30.78 |
| AT | 21.81 | 26.52 | 35.69 | 27 | 28.81 | 26.49 | 51.56 | 53.23 | 37.66 |
| AT | 34.24 | 38.31 | 30.86 | 28.27 | 32.65 | 30.26 | 49.85 | 55.59 | 35.92 |
| AT | 24.15 | 34.36 | 25.05 | 33.73 | 27.29 | 33.77 | 48.6 | 51.54 | 32.58 |
| AT | 21.96 | 38.25 | 25.49 | 29.76 | 25.84 | 39.07 | 57.42 | 49.45 | 34.71 |
| AT | 22.9 | 19.05 | 41.75 | 29.23 | 31.36 | 35.17 | 57.92 | 57.13 | 32.15 |
| AT | 41.35 | 29.3 | 36.64 | 27.88 | 31.79 | 40 | 48.29 | 59.13 | 42.36 |
| AT | 33.5 | 41.21 | 36.18 | 37.14 | 28.56 | 30.99 | 47.83 | 47.56 | 41.55 |
| AT | 28.86 | 34.08 | 36.85 | 31.48 | 38.67 | 31.87 | 52.62 | 47.65 | 37.56 |

Table S5.

Supplementary Table 5. Antibodies used in this study.

| **Antibodies** | **Species** | **application** | **Concentration** | **Company source** | **Cat #** |
| --- | --- | --- | --- | --- | --- |
| SKAP2 | Rabbit | IF/WB | 1:100/1:1000 | Affinity | DF12085 |
| hnRNPR | Rabbit | IF/RIP | 1:100 | Sigma | HPA026092 |
| hnRNPR | Rabbit | IF | 1:100 | ABclonal | A21321 |
| DDX4/MVH | Rabbit | IF | 1:400 | abcam | ab13840 |
| SYCP3 | Rabbit | IF | 1:200 | abcam | ab15093 |
| SYCP3 | Mouse | IF | 1:100 | santa | sc-74569 |
| HRP Goat anti-mouse IgG | Goat | WB | 1:10000 | FineTest | Cat# FNSA-0003 |
| HRP Goat anti-rabbit IgG | Goat | WB | 1:10000 | FineTest | Cat# FNSA-0004 |
| Dylight 488 Goat anti-mouse IgG | Goat | IF | 1:500 | Abbkine | A23210 |
| Dylight 594 Goat anti-rabbit IgG | Goat | IF | 1:500 | Abbkine | A23420 |
| Alexa FluorTM 488 Goat anti-mouse (H+L) | Goat | IF | 1:500 | Invitrogen | A11029IgG |
| Alexa FluorTM 488 Goat anti-rabbit IgG (H+L) | Goat | IF | 1:500 | Invitrogen | A32731 |
| Alexa FluorTM 594 Goat anti-rabbit IgG (H+L) | Goat | IF | 1:500 | Invitrogen | A11032 |
| m6A | Mouse | RIP | 10μg/mg | Proteintech | 68055-1-Ig |
| GAPDH | Rabbit | WB | 1:4000 | Proteintech | 10494-1-AP |
| γH2AX | Rabbit | IF | 1:200 | ABclonal | AP0099 |
| γH2AX | Rabbit | IF | 1:200 | ABclonal | AP0687 |
| α-TUBULIN | Mouse | IF | 1:100 | Bioss | bsm-33039M |
| α-TUBULIN | Rabbit | IF | 1:100 | MedChemExpress | HY-P86200 |
| F-ACTIN | Mouse | IF/WB | 1:100/1:800 | ABIN | ABIN118960 |

**ALL original and uncropped films of Western blots**

**
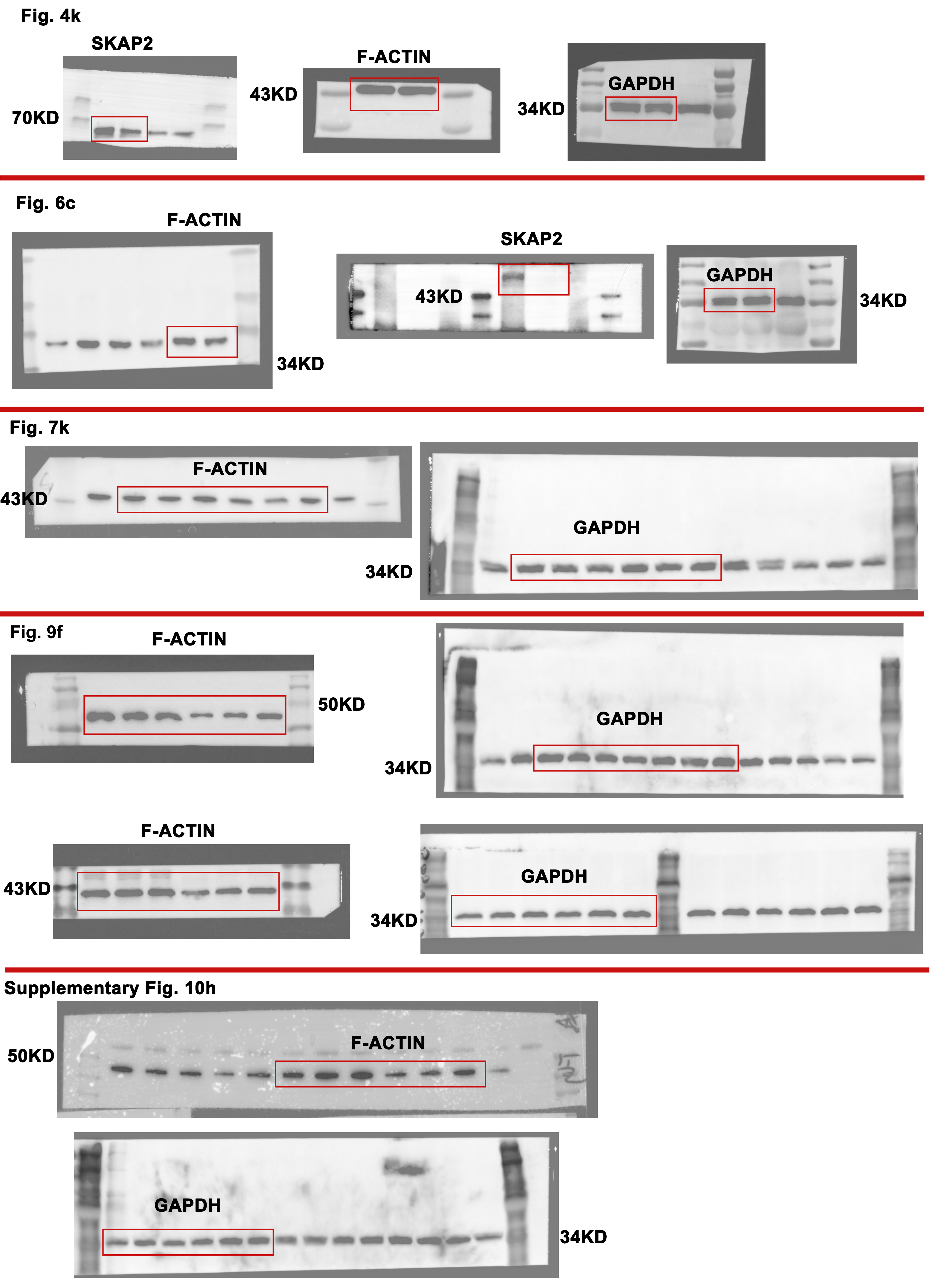
**

**ALL original and uncropped films of PCR gels**

**
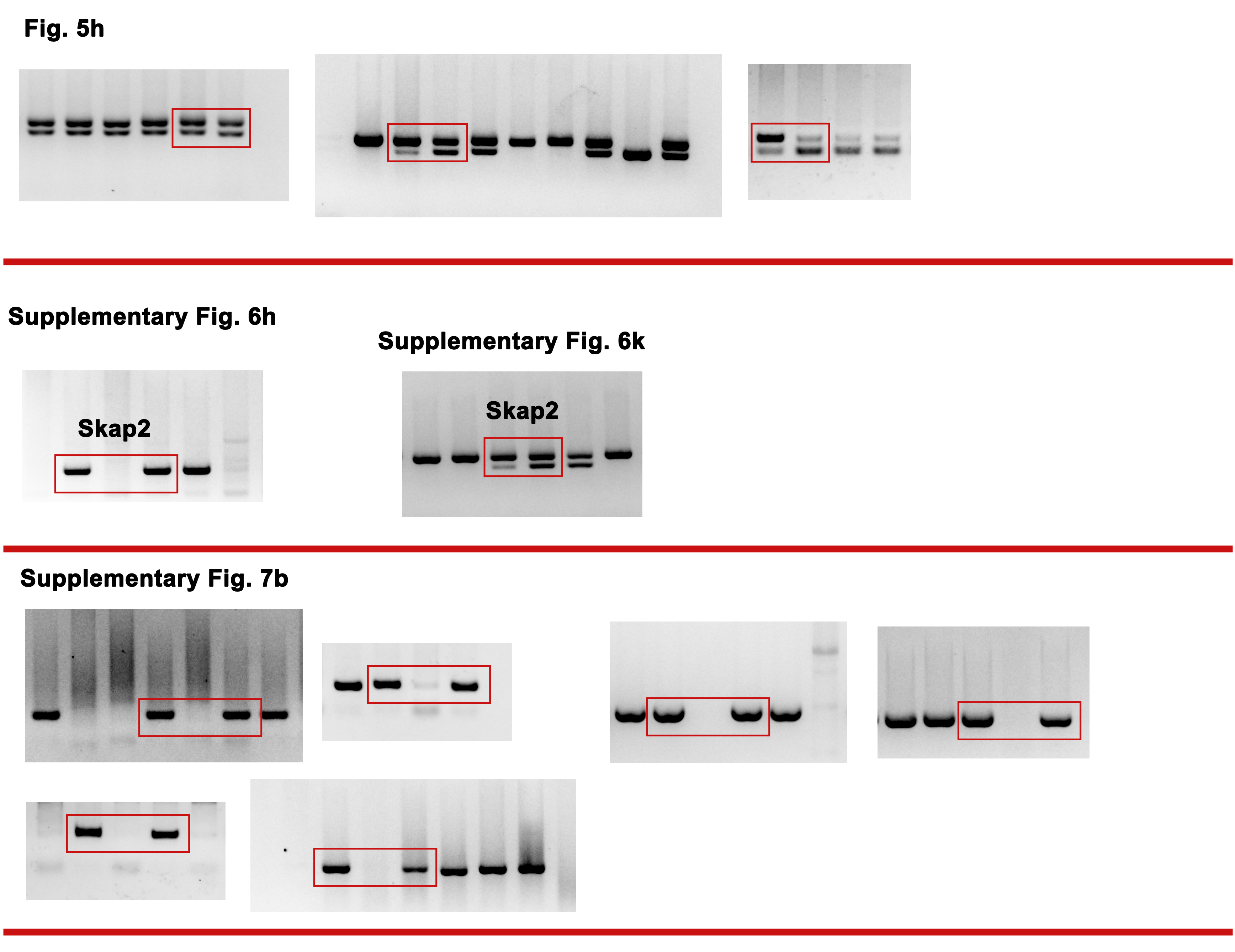
**
